# Transformation of Polysulfide Catholyte Chemistry through Lithium-Arene Complexes for Superior Solubility and Cyclability in Li–S Batteries

**DOI:** 10.1021/jacsau.5c00537

**Published:** 2025-07-09

**Authors:** Ngoc Long Le, Sih-Ling Hsu, Thi Hang Vu, Chi-You Liu, Quang Huy Dinh, Avi Arya, Elise Yu-Tzu Li, Yu-Sheng Su

**Affiliations:** † International College of Semiconductor Technology, 34914 National Yang Ming Chiao Tung University, 1001 Daxue Road, Hsinchu 300093, Taiwan; ‡ Department of Chemistry, 34879 National Taiwan Normal University, No. 88, Section 4, Tingzhou Road, Taipei 11677, Taiwan; § Department of Chemistry, Soochow University, No. 70, Linhsi Road, Taipei 11102, Taiwan; ∥ Industry Academia Innovation School, National Yang Ming Chiao Tung University, 1001 Daxue Road, Hsinchu 300093, Taiwan

**Keywords:** lithium−sulfur cells, polycyclic aromatic hydrocarbons, non-toxic synthesis, high-energy density, DFT calculations

## Abstract

The development of high-performance lithium–sulfur batteries (LSBs) has been focused on overcoming the limitations associated with traditional polysulfide catholyte synthesis. We report an innovative catholyte synthesis method using lithium-arene complexes, offering significant advancements in terms of solubility, stability, and scalability. By leveraging the interaction of metallic lithium with biphenyl (BP) and sulfur, we developed a Li+BP+S catholyte formulation that outperforms conventional Li_2_S+S systems. The Li+BP+S catholyte demonstrates superior solubility, achieving up to 12 M active sulfur and faster dissolution rates at lower temperatures, reducing preparation times by 66%. Electrochemical evaluations revealed enhanced capacity retention, with the catholyte maintaining 83.2% of its initial capacity after 500 cycles and exhibiting minimal capacity fading of 0.03% per cycle. Material characterization confirmed a uniform sulfur distribution, improved charge transfer capability, and reduced polysulfide clustering, as evidenced by nuclear magnetic resonance (NMR), scanning electron microscopy (SEM), and X-ray photoelectron spectroscopy (XPS) analyses. The Li+BP+S system also demonstrated high-rate capability and long-term stability, retaining significant capacity under lean electrolyte conditions. The mechanism by which the addition of arenes aids Li dissolution is also proposed on the basis of theoretical calculations. These findings highlight the potential of lithium–arene complexes to revolutionize LSB technology, paving the way for safer, more efficient, and scalable LSB systems.

## Graphical abstract

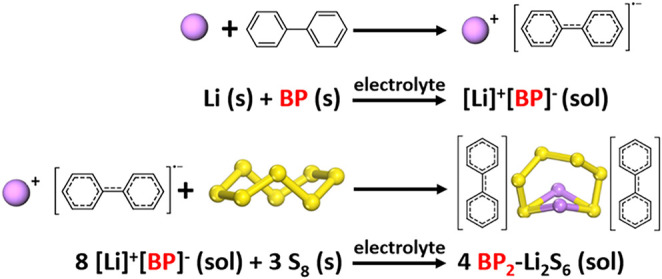

## 1 Introduction

Rechargeable lithium–sulfur batteries (LSBs) have emerged as promising alternatives for next-generation energy storage systems because of their high theoretical energy density, improved safety characteristics, and cost-effectiveness, attributed to the abundance of sulfur on Earth. Sulfur, based on its redox reaction S^0^ ↔ S^2–^, offers an outstanding theoretical capacity of 1675 mAh g^–1^.^1,2^ Combined with the standard operating voltage of 2.15 V relative to lithium metal (Li^+^/Li^0^) and the theoretical capacity of a lithium anode at 3860 mAh g^–1^, the projected energy density of LSBs is approximately 2600 Wh kg^–1^.^1,2^ This energy density is nearly seven times greater than that of conventional lithium-ion batteries, such as graphite/LiCoO_2_ batteries, which achieve a maximum energy density of approximately 387 Wh kg^–1^.^2^ Over the past decade, extensive research has been directed toward enhancing the LSB performance. A key challenge of LSB use arises from the dissolution of lithium polysulfides (LiPSs), which are intermediate species formed during sulfur redox reactions.^1^ These soluble species tend to migrate between the cathode and anode, resulting in a “shuttle effect” that compromises capacity and cycle life.^1^ To mitigate this, strategies such as the incorporation of carbon-based or other porous materials have been explored to trap soluble LiPSs through physisorption or chemisorption.^3–8^ Additionally, catalytic materials have been employed to accelerate the conversion kinetics of sulfur species, thereby increasing the reaction efficiency and suppressing undesirable side reactions.^9–12^

The electrochemical processes in LSBs are complicated. During discharge, the cathode undergoes lithiation, initially forming soluble long-chain polysulfides (e.g., Li_2_S_8_).^1^ These are subsequently reduced to shorter-chain polysulfides (Li_2_S_6_ and Li_2_S_4_) and eventually to insoluble Li_2_S_2_ or Li_2_S.^1,13^ This sequence of transformations involves the dissolution and precipitation of LiPSs, which transition between solid and solution states. The concept of a “catholyte”, where LiPSs are predissolved in the electrolyte, has been proposed to improve cathode fabrication. Fu et al. introduced a liquid catholyte using LiPSs integrated with a free-standing current collector, achieving high initial charge capacities at varying rates.^14^ The conventional synthesis of LiPSs typically involves the codissolution of Li_2_S and sulfur in an electrolyte. However, this method presents significant drawbacks: Li_2_S is air sensitive, reacts with moisture and releases toxic H_2_S gas, and it is both expensive and challenging to store, limiting its scalability. To address these issues, this study introduces a novel synthesis approach using the reaction of metallic lithium with arenes and sulfur to produce LiPS catholytes. Arenes, such as biphenyl (BP) and naphthalene (Naph), form a stable complex (LiBP or LiNaph) with lithium, which acts as a lithiation agent,^15,16^ facilitating the reaction with sulfur to generate LiPSs. This method, illustrated in Figure 1, achieves high solubility and rapid preparation of LiPS catholytes by leveraging lithium-arene complexes. The resulting catholyte offers superior capacity and enhanced cycling stability and eliminates the need for toxic Li_2_S, thereby supporting safer large-scale production and improved LSB performance. By leveraging this innovative synthesis technique, we aim to revolutionize the production of LiPS catholytes, paving the way for more efficient, scalable, and sustainable LSB technologies.

**Figure 1:**
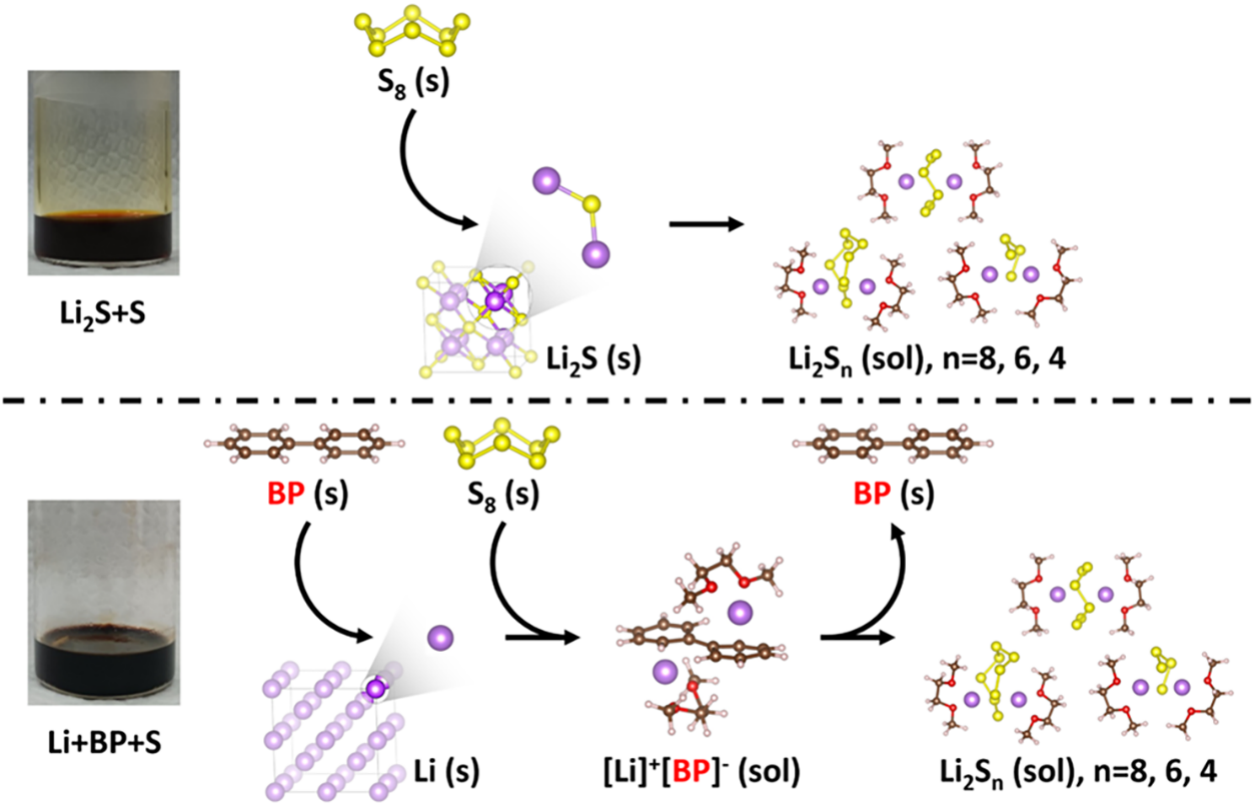
Schematic illustration of the dissolution mechanisms for (top) the conventional catholyte synthesis method using lithium sulfide and sulfur (Li_2_S+S) and (bottom) the developed method utilizing metallic lithium, biphenyl, and sulfur (Li+BP+S). The new approach achieves enhanced solubility, accelerates preparation, and eliminates the need for toxic and air-sensitive Li_2_S, enabling the safer and more scalable production of LiPS catholytes.

## 2 Experimental Section

### 2.1 Synthesis of Polysulfide Catholytes

The electrolyte was prepared by dissolving 1.85 M lithium bis­(trifluoromethanesulfonyl)­imide salt (LiTFSI; Sigma–Aldrich) and 0.1 M lithium nitrate additive (LiNO_3_; Kanto Chemical) in a 1:1 (v/v) mixture of 1,3-dioxolane (DOL; Thermo Scientific) and 1,2-dimethoxyethane (DME; Alfa-Aesar). A traditional Li_2_S+S polysulfide solution was synthesized by mixing Li_2_S powder (Alfa-Aesar) and sublimed sulfur (Showa Chemical) in a 1:5 molar ratio with the prepared electrolyte solution, as reported previously.^14^ The mixture was stirred and heated under controlled conditions until all of the solutes were completely dissolved, yielding a brown solution. The Li+BP+S polysulfide catholyte was prepared by chemically reacting metallic lithium, biphenyl (Thermo Scientific), and sublimed sulfur in a 1:1:3 molar ratio in the prepared electrolyte solution. This solution was stirred and heated under controlled conditions until all of the solutes were fully dissolved, resulting in a dark brown solution. All synthesis steps were conducted in an argon-filled glovebox, with oxygen and moisture levels consistently maintained below 0.5 ppm.

### 2.2 Cell Assembly and Electrochemical Characterization

CR2032-type coin cells were assembled by following a standardized procedure inside an argon-filled glovebox. First, the concentrated polysulfide catholyte was added onto a free-standing carbon nanotube (CNT; Taiwan Carbon Materials Corp.) electrode prepared using a previously reported CNT papermaking process.^17^ A Celgard 2400 separator was placed over the catholyte-absorbed CNT electrode, and the electrolyte solution was uniformly distributed across both sides of the separator. A lithium metal anode was then placed on top of the separator, and the cell was sealed for electrochemical testing. Symmetric cells were assembled using two identical CNT electrodes, each loaded with 20 μL of 1 M polysulfide solution and 20 μL of 1 M electrolyte, separated by a Celgard separator.

Charge/discharge profiles, rate performance, and cycling stability were evaluated by using a programmable battery cycler (Neware CT4008). After assembly, the cells were allowed to rest for 12 h before testing. The initial cycling involved three formation cycles of discharging to 1.8 V and charging to 2.8 V at 0.1C, followed by cycling at 0.5C (1C = 1675 mA g^–1^). Cyclic voltammetry (CV) and electrochemical impedance spectroscopy (EIS) measurements were performed by using a potentiostat (BioLogic SP-50e). CV scans for LSBs were recorded between 1.8 and 2.8 V at a scan rate of 0.1 mV s^–1^, whereas symmetrical cells were scanned between −1.0 and 1.0 V at scan rates ranging from 5 to 20 mV s^–1^. EIS measurements were conducted over a frequency range of 1 MHz to 10 mHz with a 10 mV amplitude, and resistance values were extracted from Nyquist plots by using EC-Lab software.

### 2.3 Materials Characterization

Ultraviolet–visible spectroscopy (UV–Vis; Jasco V-370) was employed to analyze the LiPS species in the catholyte solutions. ^7^Li NMR analysis of the catholyte was performed by using a JEOL ECZ500R/S1 NMR500 spectrometer. Raman spectroscopy (Horiba LabRam HR800) was used to characterize the cycled electrodes, with a scanning range of 100–3000 cm^–1^. Crystallinity was assessed using X-ray diffraction (XRD; Bruker D8) with Cu Kα radiation, covering a range of 10 to 60°. Microstructural analyses were carried out by scanning electron microscopy (SEM) and transmission electron microscopy (TEM; JEOL JEM-2100F), both of which are equipped with energy-dispersive X-ray spectroscopy (EDS) detectors for elemental mapping. Depth profile compositional changes after cycling were investigated by using X-ray photoelectron spectroscopy (XPS; ULVAC-PHI Quantera II) with argon ion etching at 1 kV.

## 3 Results and Discussion

### 3.1 Polysulfide Solubility and Dissolution Rate Assessments

The preparation of Li+BP+S polysulfide solutions is highly sensitive to the addition sequence of lithium metal, BP, and sulfur. As shown in Figure S1, initially mixing Li+S, followed by the addition of BP, does not promote polysulfide dissolution. Conversely, starting with either Li+BP or BP+S and subsequently adding the third component effectively facilitate polysulfide dissolution. This observation implies that fresh lithium must not come into direct contact with sulfur initially, as this interaction hinders the reaction between lithium and BP necessary for forming the LiBP complex ion pair.

To evaluate the solubility limits of Li+BP+S and Li_2_S+S polysulfide solutions, we conducted experiments to dissolve each type of polysulfide in different electrolytes with varying lithium salt concentrations. In an electrolyte solvent containing 1.85 M LiTFSI salt, Li+BP+S polysulfide achieves a maximum solubility of 4 M active sulfur, whereas Li_2_S+S polysulfide reaches only 1.5 M (Figure 2a). When the LiTFSI salt concentration is reduced to 1 and 0 M, the polysulfide solubility increases significantly. Notably, the solubility of Li+BP+S remains consistently higher than that of Li_2_S+S, achieving values of 8.5 and 12 M compared with 6.5 and 10 M for Li_2_S+S in 1 and 0 M electrolytes, respectively (Figures 2b and S2). These findings highlight the advantages of the Li+BP+S system in enhancing the solubility, which is crucial for enhancing active material utilization in LSBs.

**Figure 2:**
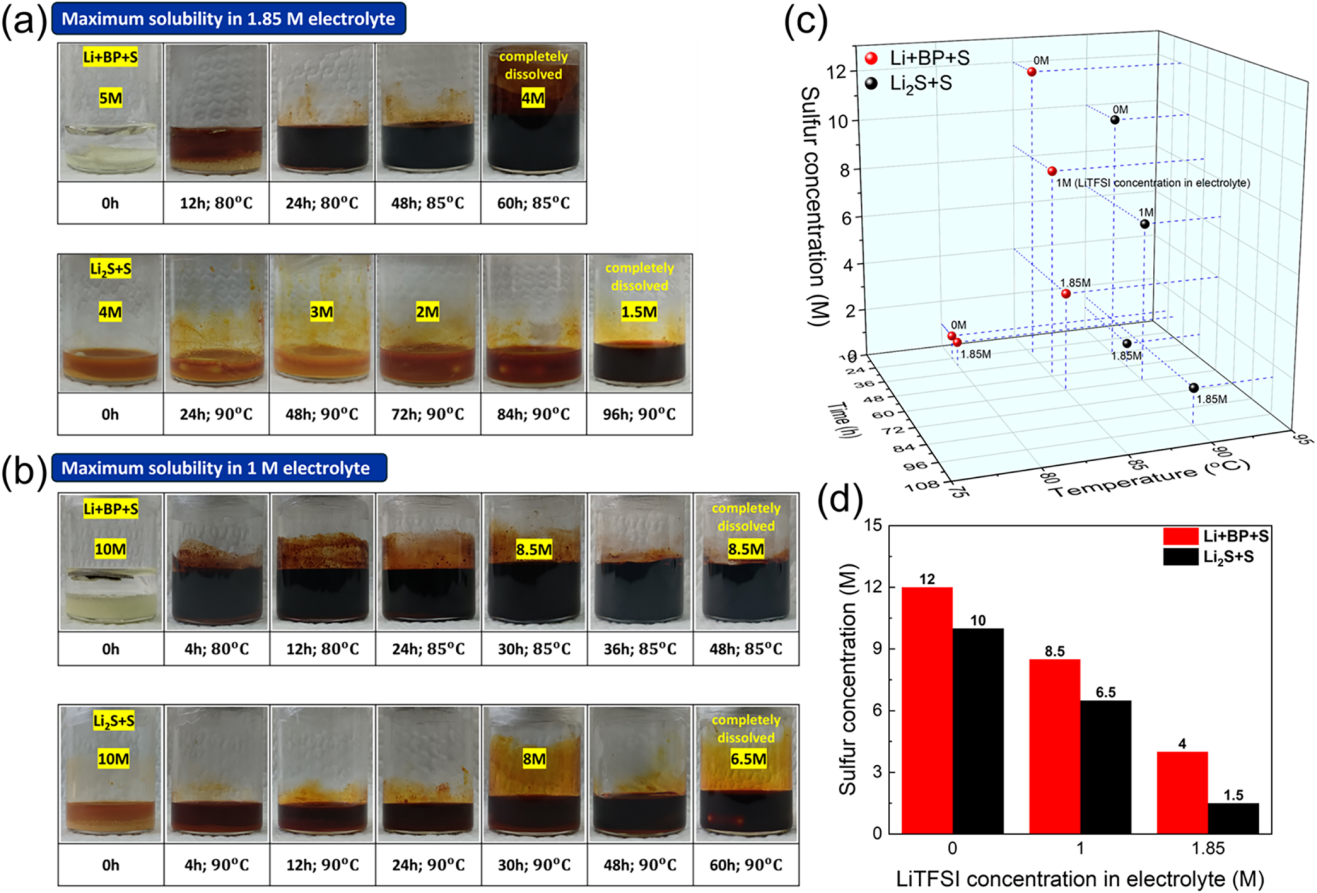
Maximum solubility of polysulfide solutions in (a) 1.85 and (b) 1 M electrolytes. (c) Correlations between time, temperature, and sulfur concentration for polysulfide solutions. (d) Maximum solubility of polysulfide in electrolytes with varying LiTFSI salt concentrations.

Figure S3 compares the dissolution rates of the two polysulfide formulations. Sulfur particles in the Li_2_S+S solution dissolve with great difficulty, even when the temperature is increased to 85 °C and the solution is stirred for 36 h. Complete dissolution requires continuous stirring for 48 h at 90 °C. In contrast, the Li+BP+S polysulfide solution achieves complete dissolution within 30 h at 80 °C. This difference highlights the role of BP in facilitating faster and more efficient dissolution at lower temperatures, making the Li+BP+S system a more practical option for catholyte preparation. Furthermore, Figure S4 shows that increasing the BP concentration significantly reduces the preparation time. For example, increasing the BP concentration from 0.5 BP (1Li + 0.5BP + 3S) to 4 BP (1Li + 4BP + 3S) decreases the dissolution time from 36 to 12 h at 80 °C. Additionally, higher BP concentrations (e.g., 1Li + 2BP + 3S) increase the maximum solubility of active sulfur to 6 M in a 1.85 M electrolyte (Figure S5). These results highlight the tunability of the Li+BP+S system, yielding both a reduced processing time and increased solubility.

Figure 2c,2d summarize the solubility and preparation times/temperatures of each polysulfide solution. Compared with Li_2_S+S, Li+BP+S consistently requires less time and lower temperatures for fabrication. Despite the more demanding fabrication process, Li_2_S+S achieves a lower solubility than Li+BP+S does, which consistently results in superior solubility across all LiTFSI concentrations. In summary, the optimized preparation condition for a concentrated Li+BP+S polysulfide solution involves the use of a molar ratio of 1Li+2BP+3S, achieving up to 6 M sulfur solubility in a 1.85 M electrolyte, with complete dissolution attained at 80 °C. This condition offers a favorable balance between solubility, processing time, and temperature, making it optimal for catholyte preparation in practical applications. The high solubility of Li+BP+S polysulfide provides sufficient active sulfur for electrochemical processes, including sulfur/lithium sulfide generation and absorption during charge–discharge cycles in LSBs. This leads to improved energy efficiency, better utilization of active materials, and enhanced battery stability and longevity.

### 3.2 Material Characterization of Polysulfide Catholytes

UV–vis spectroscopy was used to evaluate the dissolved species of the Li+BP+S and Li_2_S+S polysulfide systems. As shown in Figure 3a, both polysulfides exhibit distinct peaks corresponding to S_6_^2–^/S_4_^2–^ (315–340 nm), S_4_^2–^ (405 nm), S_8_^2–^/S_6_^2–^ (475 nm), and S_3_^•–^ (620 nm).^18–20^ In the Li+BP+S solution, there is an increased presence of S_8_^2–^/S_6_^2–^ and a reduced presence of S_3_^•–^, suggesting that the equilibrium shifts toward S_8_^2–^/S_6_^2–^ speciation. The elemental sulfur undergoes two-electron reduction (eq 1), followed by disproportionation and rapid dissociation into S_6_^2–^ and S_3_^•–^, as described in eqs 2 and 3. Additionally, S_8_^2–^ and S_6_^2–^ reduction and disproportionation may occur via eqs 4–([Disp-formula eq6])^18–22^

1 $$S_{8}+2e^{-}\to S_{8}^{2-}$$

2 $$S_{8}^{2-}\to S_{6}^{2-}+\frac{1}{4}S_{8}$$

3 $$S_{6}^{2-}\to 2S_{3}^{•-}$$

4 $$S_{8}^{2-}+2e^{-}\to 2S_{4}^{2-}$$

5 $${2S}_{6}^{2-}+2e^{-}\to 3S_{4}^{2-}$$

6 $$S_{6}^{2-}\to S_{4}^{2-}+\frac{1}{4}S_{8}$$

**Figure 3:**
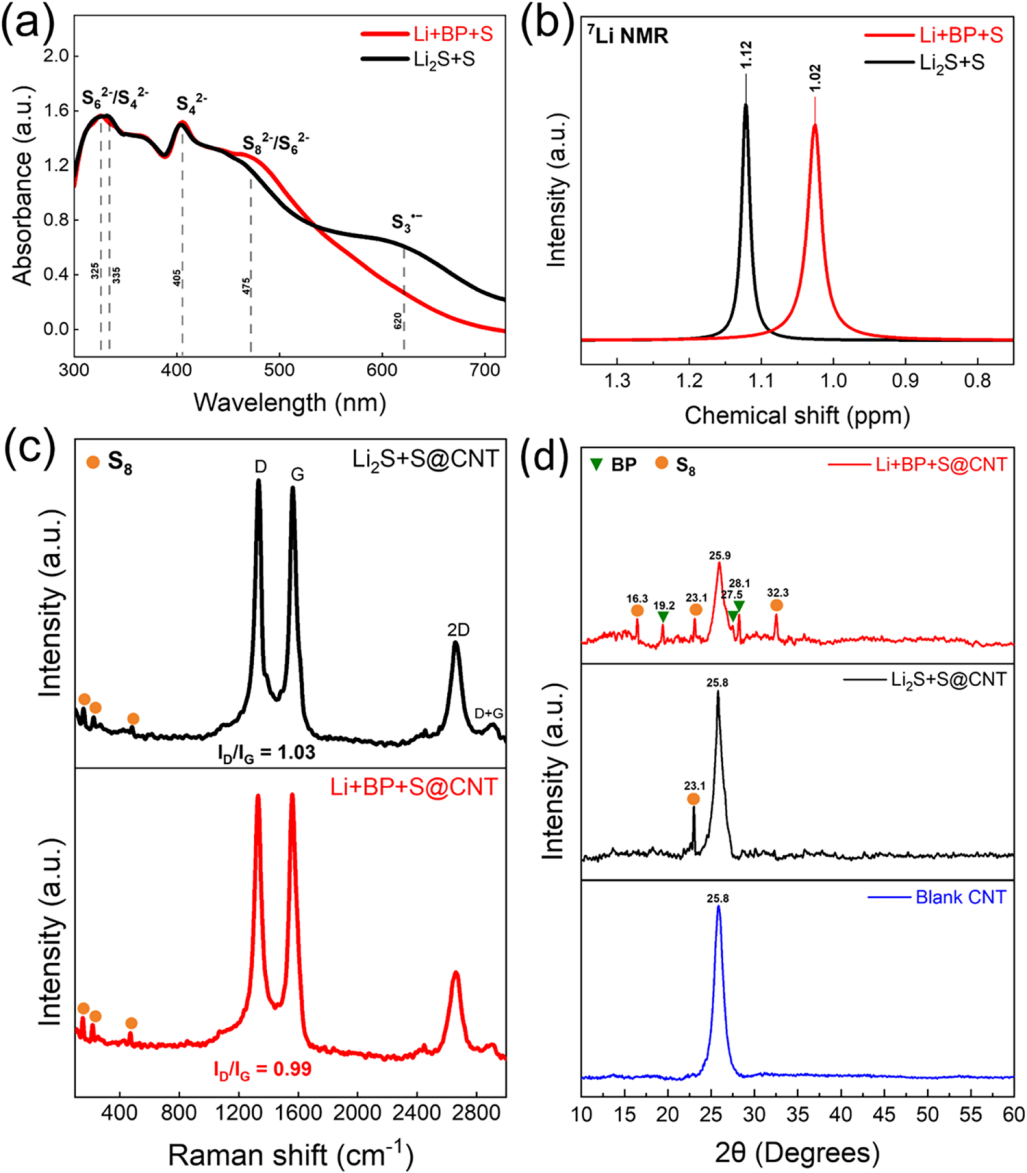
(a) UV–Vis spectra and (b) ^7^Li NMR spectra of Li+BP+S and Li_2_S+S polysulfide solutions. (c) Raman spectra of the Li_2_S+S@CNT and Li+BP+S@CNT electrodes after the 100th cycle. (d) XRD patterns of the Li+BP+S@CNT and Li_2_S+S@CNT electrodes after the 100th cycle.

These species are further reduced to lower-order polysulfides. The S_3_^•–^ radical acts as an intermediate (eqs 7 and 8), ultimately leading to solid Li_2_S formation (eqs 9 and 10)^18,21,22^

7 $$S_{3}^{•-}+e^{-}\to S_{3}^{2-}$$

8 $$2S_{3}^{•-}+2S_{3}^{2-}\to 3S_{4}^{2-}$$

9 $$S_{4}^{2-}+2e^{-}\to 2S_{2}^{2-}$$

10 $$S_{2}^{2-}+2e^{-}\to 2S^{2-}$$

The breakdown of S_3_^•–^ (eq 3) is critical for this mechanism. In the Li_2_S+S polysulfide solution, the noticeable concentration of S_3_^•–^ suggests a quick breakdown, whereas in Li+BP+S, the abundance of S_8_^2–^ and S_6_^2–^ indicates a shift in the equilibrium of eq 3. These findings suggest that the conversion mechanism of Li+BP+S polysulfides may differ from that of traditional Li_2_S+S formulations during discharge in a lean electrolyte LSB setup. While S_3_^•–^ may act as a reactive intermediate assisting in reduction,^18,21,22^ it does not remain a dominant species during the discharge process in the Li+BP+S system.

To investigate the lithium bonding in polysulfide solutions, we conducted ^7^Li NMR spectroscopy. The ^7^Li NMR spectra confirmed that BP molecules effectively inhibited LiPS clustering by forming stable BP–Li_2_S_6_ complexes, facilitating Li^+^ ion transport from the electrolyte to the LiPS chains.^23^ As shown in Figure 3b, the spectra for the Li_2_S+S and Li+BP+S polysulfides exhibit peaks at 1.12 and 1.02 ppm, respectively. The introduction of BP results in an upfield shift of approximately 0.1 ppm, indicating enhanced electron shielding around Li^+^ ions due to the distinct BP–Li_2_S_6_ coordination environment.^23^ Additionally, the Li+BP+S spectrum is broader than the Li_2_S+S spectrum, which is attributed to the reduced lithium-ion mobility resulting from stronger BP–Li_2_S_6_ interactions rather than polysulfide clustering.^24^ The observed peak broadening results from reduced molecular reorientation rates, leading to shorter T_2_ relaxation times (spin–spin relaxation).^24^ Thus, the chemical shift and peak broadening in ^7^Li NMR data serve as an effective descriptor for quantifying the interaction strength between LiPS catholytes and characterizing their lithium bonding environments.

Figure 3c shows the Raman spectra of the Li_2_S+S@CNT and Li+BP+S@CNT electrodes after 100 cycles, revealing notable peaks corresponding to sulfur and CNTs. The D peak at 1335 cm^–1^, associated with sp^3^ hybridized carbon atoms, indicates disordered defects in the CNTs, whereas the G peak at 1566 cm^–1^, linked to sp^2^ hybridized carbon atoms, reflects the vibrational properties of the carbon.^25^ The peak at 2665 cm^–1^ corresponds to the 2D band of graphene.^26^ The *I*_D_/*I*_G_ ratio is greater for Li_2_S+S@CNT than for Li+BP+S@CNT, indicating slightly fewer defects with the addition of BP in the Li+BP+S@CNT sample. Both electrodes exhibit sulfur signals at approximately 155, 219, and 477 cm^–1^ after cycling.^27^ These fully charged electrodes show weak peaks at approximately 2449 cm^–1^, corresponding to LiPSs produced during the Li–S reaction.^27^

XRD analysis (Figure 3d) revealed the crystal structures of carbon, sulfur, Li_2_S, and BP in the electrodes. For the Li+BP+S@CNT electrode, the 2θ peaks at 16.3, 23.1, and 32.3° correspond to orthorhombic α-sulfur reflections at (113), (222), and (044), respectively.^28,29^ BP peaks appear at 19.2, 27.5, and 28.1°.^30,31^ The Li2S+S@CNT electrode has fewer sulfur peaks, reflecting less efficient sulfur conversion. For all electrodes, the pronounced 2θ peak at 25.9° corresponds to (002) reflections from graphitic CNT layers (JCPDS card no. 75–1621).^32^ The CNT signal is reduced due to sulfur coverage, with the weakest signal in the Li+BP+S electrode, indicating more uniform and thicker sulfur deposition.

### 3.3 Electrochemical Evaluation of Polysulfide Catholytes

Galvanostatic charge–discharge (GCD) tests were conducted to assess the capacity of cells containing different polysulfide catholytes. Figure 4a,b present the first, 50th, 200th, and 500th cycle charge–discharge profiles at 0.5C for LSBs with Li+BP+S and Li_2_S+S catholytes. Both curves exhibit characteristic charge and discharge plateaus of LSBs. A detailed analysis of the discharge curves for the Li+BP+S and Li_2_S+S catholytes is shown in Figure 4c,d, respectively. Both catholytes demonstrate two distinct discharge stages: the first plateau near 2.25 V corresponds to the conversion of sulfur into LiPSs (Li_2_S*_n_*, where *n* ranges from 4 to 8), whereas the second plateau at approximately 2.05 V is associated with the formation of insoluble Li_2_S_2_ and Li_2_S.^13,33^ For the Li+BP+S catholyte, the initial discharge capacity at the upper plateau after the formation cycles reaches 415.3 mAh g^–1^, corresponding to over 99% of its theoretical maximum (419 mAh g^–1^).^17^ This indicates nearly complete sulfur dissolution and utilization during the first cycle. Remarkably, after 500 cycles, the upper plateau capacity remains high at 339.2 mAh g^–1^, retaining 81.7% of the initial capacity. In contrast, the Li_2_S+S catholyte has a lower initial discharge capacity of 343.9 mAh g^–1^ (82% of the theoretical value) and decays to 247.4 mAh g^–1^ after 500 cycles.

**Figure 4:**
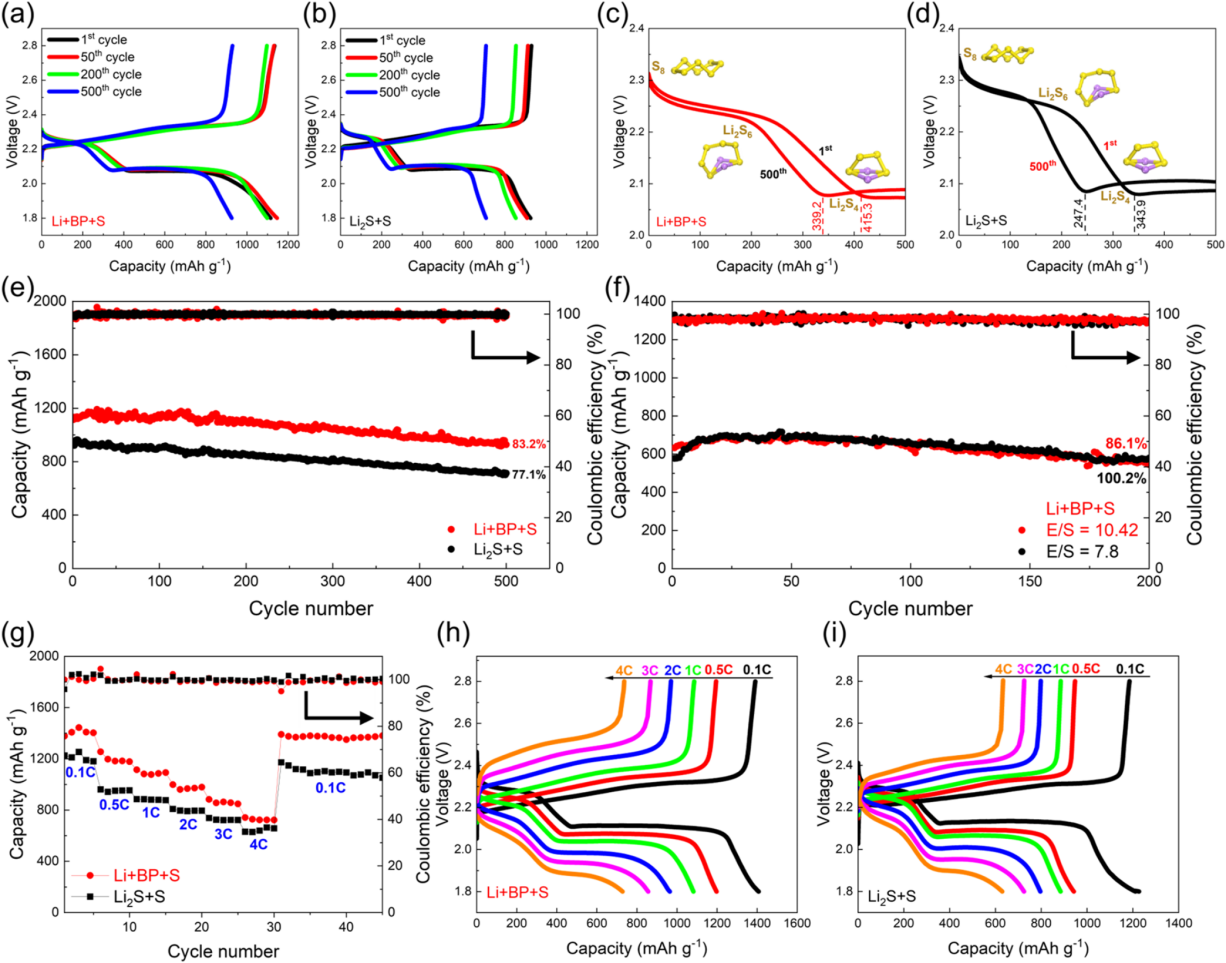
GCD profiles of (a) Li+BP+S and (b) Li_2_S+S polysulfide catholytes at a 0.5C rate after the formation cycle. Discharge profiles of (c) Li+BP+S and (d) Li_2_S+S polysulfide catholytes at various cycle numbers after the formation cycle. (e) Cycling performance of Li+BP+S and Li_2_S+S polysulfide catholytes at 0.5C. (f) Cycling performance of Li+BP+S polysulfide catholytes at 0.1C with different electrolyte-to-sulfur (E/S) ratios. (g) Rate performance of Li+BP+S and Li_2_S+S polysulfide catholytes across varying current densities. GCD profiles of (h) Li+BP+S and (i) Li_2_S+S polysulfide catholytes at different C rates.

The cycling performance at 0.5C for the Li+BP+S and Li_2_S+S catholytes is illustrated in Figure 4e, with the electrolyte-to-sulfur (E/S) ratio fixed at 31.25 μL mg_S_^–1^. Both catholytes display good electrochemical stability when paired with a CNT current collector.^14,34^ The Li+BP+S catholyte achieves an initial discharge capacity of 1115.5 mAh g^–1^ and maintains 927.8 mAh g^–1^ after 500 cycles, with minimal capacity fading of 0.03% per cycle and nearly 100% Coulombic efficiency. In comparison, the Li_2_S+S catholyte has an initial capacity of only 925.1 mAh g^–1^, which decreases to 713.3 mAh g^–1^ after 500 cycles, corresponding to a reduction in degradation rate of 0.046% per cycle, indicating inferior cycling stability. To evaluate the impact of catholyte formulation modifications, biphenyl was replaced with naphthalene to form a Li+Naph+S catholyte. This system maintains comparable cycling stability, albeit with a lower specific capacity (Figure S6). Additionally, the long-term stability of the Li+BP+S polysulfide solutions was assessed by assembling cells up to 30 days after catholyte preparation (Figure S7). The Li+BP+S catholyte retained a high capacity and exhibited excellent stability, highlighting its durability. To demonstrate the practical viability of the Li+BP+S catholyte for high-energy-density LSBs, cells were assembled with low E/S ratios.^35,36^ The corresponding sulfur areal loadings were 1.45 mg cm^–2^ for E/S = 10.42 μL of mg_S_^–1^ and 2.17 mg cm^–2^ for E/S = 7.8 μL of mg_S_^–1^, allowing evaluation under practically relevant conditions. As shown in Figure 4f, the cells achieve initial discharge capacities of 633.1 and 577.8 mAh g^–1^, respectively. After 200 cycles, these capacities stabilize at 544.8 and 579.2 mAh g^–1^, with the cell at an E/S ratio of 7.8 μL mg_S_^–1^ retaining over 100% capacity, showcasing outstanding stability with reduced electrolyte usage.

Rate performance tests for both catholytes, conducted across varying current densities (0.1–4 C and back to 0.1C), are displayed in Figure 4g. The Li+BP+S catholyte demonstrates superior rate capability, achieving a capacity of 742.3 mAh g^–1^ at 4C and recovering to 1,391.1 mAh g^–1^ when the current is returned to 0.1C, highlighting its efficient sulfur utilization and excellent electrochemical reversibility. Although increased voltage polarization is observed in the Li+BP+S system, as compared in Figure 4h,4i, its higher solubility significantly improves the availability of active LiPSs, thereby contributing to its superior electrochemical performance. In contrast, the Li_2_S+S catholyte exhibited a lower capacity of 631.2 mAh g^–1^ at 4C, reflecting a limited rate performance.

To provide a broader perspective on the performance of our catholyte formulation, a benchmarking summary has been included in Table S1, which compares the Li+BP+S system with those of other reported polysulfide catholytes. Our system shows comparable or even better capacity retention and cycling stability, even under low E/S ratios, and does so using a simple CNT paper current collector without any functional catalysts or engineered interlayers. Given that few new polysulfide catholyte formulations have been reported in recent years, the performance of the Li+BP+S system highlights its strong potential to advance LSB development.

Figure 5a shows the initial CV curves of the two catholytes, revealing two cathodic peaks at ∼2.2 and ∼2.0 V. These peaks correspond to the transformation of sulfur into higher-order LiPSs (Li_2_S*_n_*, *n* ≥ 4) and their subsequent reduction to Li_2_S_2_ and Li_2_S.^36,37^ For the Li+BP+S catholyte, a slightly lower reaction voltage of 1.91 V is observed for solid-phase sulfur conversion. This behavior is likely due to the greater quantity of deposited active species in the system, which can introduce localized mass transport limitations and additional nucleation barriers for Li_2_S_2_/Li_2_S formation rather than indicating slower intrinsic kinetics. This interpretation is supported by the significantly larger CV peak areas and the extended upper discharge plateau observed in Figure 4c,d, suggesting more complete active sulfur utilization in the Li+BP+S system. During the anodic scan, the peaks at 2.35 and 2.45 V indicate the reconversion of lower-order LiPSs back to elemental sulfur,^36,37^ which is consistent with the GCD profiles in Figure 4a,4b. Figure 5b,5c depict the CV curves over the first four cycles for the Li+BP+S and Li_2_S+S catholytes, respectively. The higher peak currents in the Li+BP+S catholyte suggest enhanced active sulfur utilization and additional capacity contributions. Symmetrical cell CV tests (Figure 5d–f) revealed stronger and broader peaks in the Li+BP+S catholyte, indicative of more complete redox reactions and efficient catalytic transformation of LiPSs.^36^

**Figure 5:**
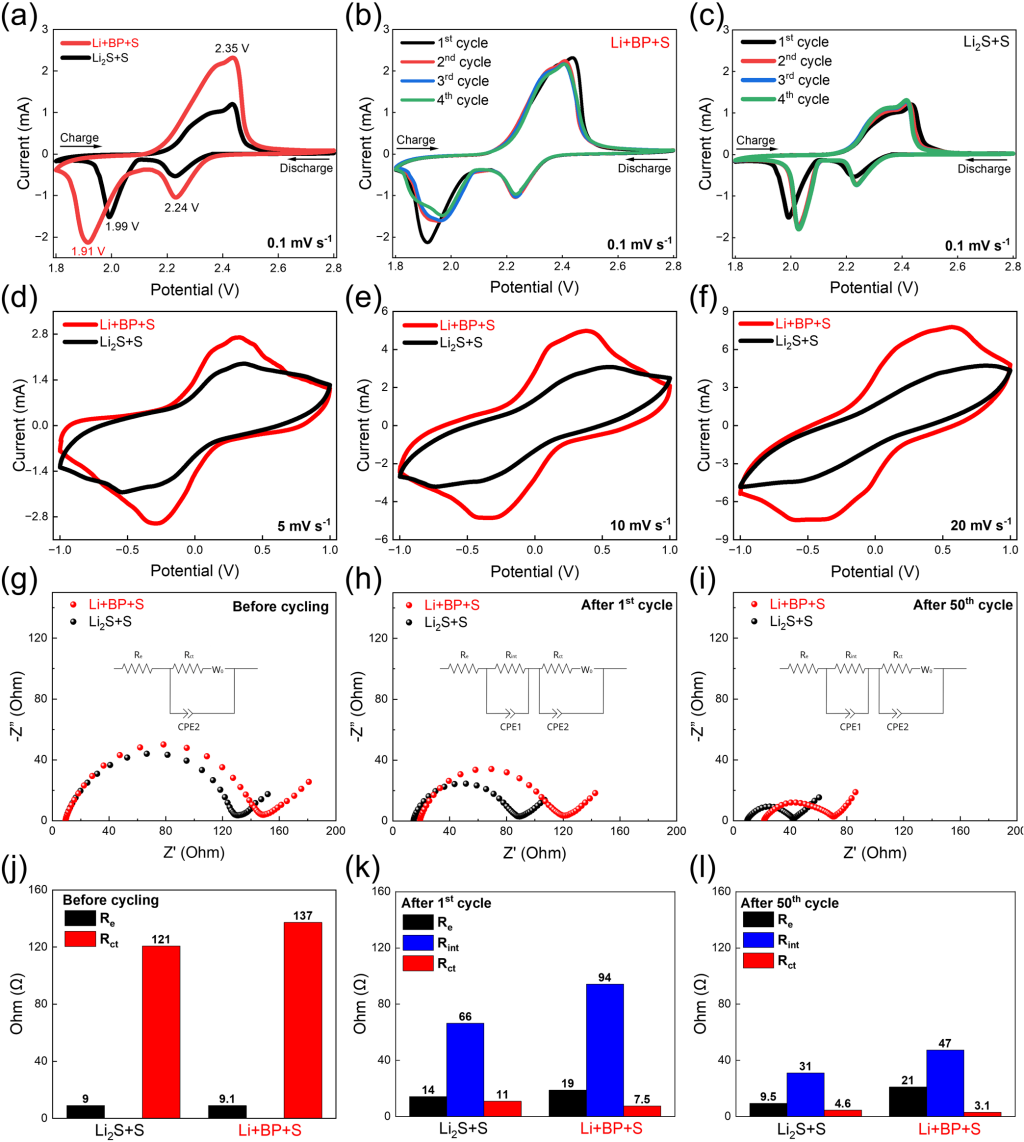
(a) Comparative CV plots of Li+BP+S and Li_2_S+S polysulfide catholytes. Individual CV profiles of (b) Li+BP+S and (c) Li_2_S+S polysulfide catholytes at a scan rate of 0.1 mV s^–1^. CV curves of symmetric cells with Li+BP+S and Li_2_S+S polysulfide catholytes at scan rates of (d) 5, (e) 10, and (f) 20 mV s^–1^. (g–i) Nyquist plots comparing the EIS results of Li+BP+S and Li_2_S+S polysulfide catholytes at different cycle numbers (inset: equivalent circuit model). (j–l) Impedance comparisons for Li_2_S+S and Li+BP+S polysulfide catholytes showing electrolyte resistance (*R*_e_), interfacial resistance (*R*_int_), and charge transfer resistance (*R*_ct_) across cycles.

EIS data for the two catholytes at different cycles are shown in Figure 5g–i. Nyquist plots feature a high-to-midfrequency semicircle and a low-frequency linear segment. Initially, the charge transfer resistance (*R*_ct_) of the Li+BP+S catholyte (137 Ω) is slightly greater than that of the Li_2_S+S catholyte (121 Ω) (Figure 5j),^36,38^ likely because the presence of BP increases the charge transfer resistance. Upon cycling, both systems show a significant reduction in *R*_ct_ (Figure 5k,5l), which is attributed to smooth polysulfide migration leading to optimized active sulfur deposition. However, the *R*_ct_ decrease is more pronounced for the Li+BP+S catholyte, reflecting its uniform distribution of active sulfur species within the electrode. With extended cycling, the Li+BP+S catholyte shows a slight increase in electrolyte resistance (*R*_e_) due to higher ion concentrations and viscosity,^36,38^ resulting from greater LiPS dissolution. Additionally, the interfacial resistance (*R*_int_), influenced by the deposition of solid sulfur/lithium sulfide films,^23^ is higher for the Li+BP+S catholyte because of its greater active sulfur utilization.

### 3.4 Polysulfide Catholyte Postcycling Analysis

To examine the morphology and surface structure of the electrodes, SEM imaging was conducted. Figure 6a,6d,g show SEM images of blank CNTs (fresh state), Li_2_S+S@CNT (after 100 cycles), and Li+BP+S@CNT (after 100 cycles), respectively. The pristine CNT electrode (Figure 6a) exhibited an intertwined network of CNT fibers, forming a conductive framework. After 100 cycles, significant morphological changes are observed, such as sulfur deposition on the CNT surface (Figure 6d,6g). In the case of Li_2_S+S@CNT (Figure 6d,6e), sulfur clustering is pronounced, leading to uneven particle agglomeration. In contrast, the SEM and EDS data for the Li+BP+S@CNT electrode (Figure 6g,6h) reveal a uniform sulfur distribution across the CNTs, with no observable sulfur clusters. This homogeneous sulfur dispersion is critical for enhancing sulfur utilization and, consequently, improving the capacity of LSBs. Cross-sectional SEM images of the blank CNT, Li_2_S+S@CNT, and Li+BP+S@CNT electrodes are shown in Figure 6c,6f,6i, respectively. The thickness of the fresh CNT electrode is approximately 25.5 μm, whereas after 100 cycles, the thickness increases to 44.1 μm for Li_2_S+S@CNT and 56.5 μm for Li+BP+S@CNT. The greater thickness of the Li+BP+S@CNT electrode indicates better active sulfur conversion, resulting in more deposition and, consequently, a greater capacity contribution.

**Figure 6:**
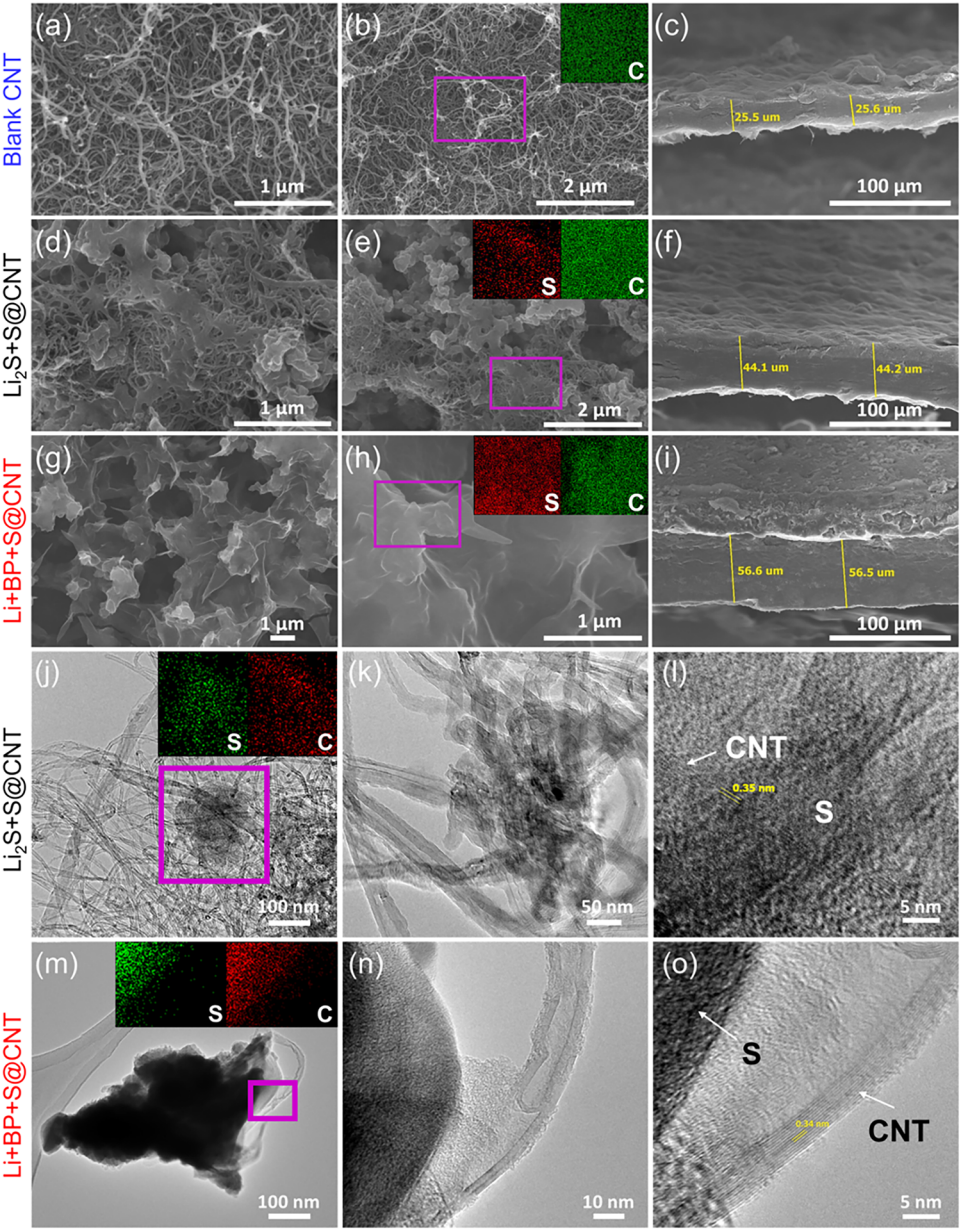
SEM, EDS mapping, and cross-sectional SEM images of (a–c) blank CNTs, (d–f) Li_2_S+S@CNT, and (g–i) Li+BP+S@CNT electrodes after 100 cycles. TEM, EDS mapping, and HRTEM images of the (j, l) Li_2_S+S@CNT and (m, o) Li+BP+S@CNT electrodes after 100 cycles.

To gain deeper insights into the microstructure and crystallinity of the cycled electrodes, TEM imaging was conducted. As shown in Figure 6j, the Li_2_S+S@CNT electrode exhibited a conductive CNT fiber network with sparsely distributed sulfur particles, reflecting an uneven sulfur distribution. In contrast, the Li+BP+S@CNT electrode (Figure 6m) exhibited a dense and uniform deposition of sulfur, which was consistent with the SEM findings. The CNTs were readily identified by their one-dimensional morphology and lattice fringes (∼0.34 nm) observed in the high-resolution TEM (HRTEM) images (Figure 6l,o), corresponding to the (002) plane of graphitic carbon.^39^ Amorphous and darker contrast regions associated with sulfur species were distinguished by EDS sulfur signals in the mapping images (Figure 6j,m), despite the lack of clear lattice fringes.

To analyze the chemical state changes in the electrodes after cycling, XPS depth profiling was conducted for the blank CNT substrate and cycled Li_2_S+S@CNT and Li+BP+S@CNT electrodes (Figure S8). The Ar^+^ beam was used for material etching, with sputtering intervals of 0 to 5 min at a rate of 15.9 nm min^–1^ (based on a SiO_2_ standard). XPS C 1s spectra (Figure S8a,c) show minimal variation in peak intensities and positions across the layers for all samples. The deconvoluted C 1s spectra (Figure S9) reveal a prominent peak at ∼284.5 eV, attributed to sp^2^ C–C bonding.^36,40^ Compared with those of the blank CNTs, the sulfur species covering the surface of the Li_2_S+S@CNT and Li+BP+S@CNT electrodes reduced the C–C bonding signal intensity. Similar trends without significant differences are observed in the XPS Li 1s spectra (Figure S8d–e), with peaks corresponding to Li–S and Li–O bonds at ∼55.3 and ∼56.3 eV, respectively (Figure S10).^36,41,42^ Notably, the Li–S bonds are concentrated on the surface of the Li_2_S+S@CNT electrode but are uniformly distributed in Li+BP+S@CNT, indicating better LiPS penetration and distribution in the latter.

For the sulfur analysis, the XPS S 2p spectra revealed negligible sulfur signals for the blank CNTs due to the lack of catholyte addition (Figure S8f). In Li_2_S+S@CNT (Figure S8g), the signals at ∼162 eV decrease rapidly from the surface to the core, indicating a surface-concentrated active sulfur species. Conversely, in Li+BP+S@CNT (Figure S8h), the sulfur signals increase from the surface to the core, demonstrating a uniform sulfur distribution. The fitted S 2p spectra (Figure 7a,7b) exhibit peaks at ∼160 eV, corresponding to Li_2_S, as well as peaks for Li_2_S_2_ (∼161.8 eV), polysulfides (PS, ∼163 eV), and S_8_ (∼164.4 eV).^38,40^ The weak S_8_ signals suggest the presence of minimal sulfur on the surface, indicating that charged species are located primarily within the electrode. Oxidized sulfur species, such as sulfite ([SO_3_]^2–^) and lithium thiosulfate ([S–SO_3_]^2–^), are observed at ∼165.7–167.2 eV, likely arising from LiTFSI and LiNO_3_ reduction products.^43^ The broad peak at ∼168.7–170.4 eV corresponds to sulfate ([SO_4_]^2–^) species, which are likely derived from side reactions during cycling. Since Figure 7a,7b highlight the distinct sulfur distributions in the cycled Li_2_S+S@CNT and Li+BP+S@CNT electrodes, Figure 7c illustrates the surface-to-core distributions of the active sulfur species.^38^ In Li_2_S+S@CNT, Li_2_S_2_ and PS are concentrated on the surface but diminish toward the core, whereas S_8_ is sparse on the surface but concentrated in the core, indicating uneven sulfur deposition. This distribution can hinder charge transfer due to the large concentration gradients of sulfur species. In contrast, Li+BP+S@CNT results in a uniform distribution of Li_2_S_2_, S_8_, PS, and even Li_2_S (albeit in small amounts, as the sample is in the charged state) throughout the electrode. This balanced sulfur distribution facilitates efficient charge transfer and maximizes active sulfur utilization, resulting in a higher capacity and superior cycling stability.

**Figure 7:**
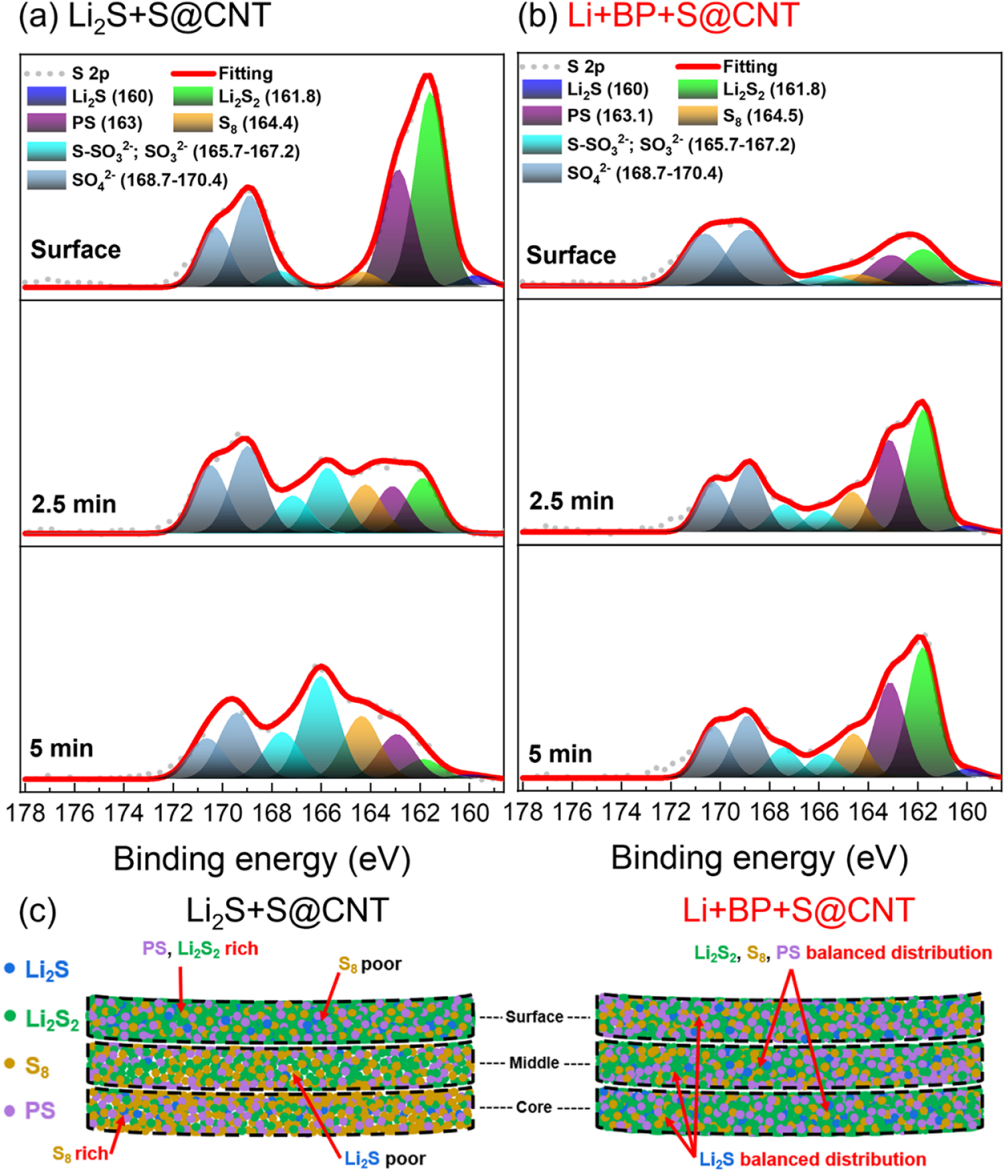
XPS spectra of the S 2p peak for the (a) Li_2_S+S@CNT and (b) Li+BP+S@CNT electrodes after 100 cycles, along with their fitted peaks. (c) Schematic illustration of the active sulfur species distribution within the Li_2_S+S@CNT and Li+BP+S@CNT electrodes based on peak area analysis.

### 3.5 Simulation of Arene-Aided Lithium Dissolution

To further elucidate the role of arenes in lithium dissolution and polysulfide formation, we performed density functional theory (DFT) simulations to investigate the interactions among lithium, solvent molecules, and arene additives. Figure S11 shows the optimized geometries and binding energies (*E*_b_) of different combinations of Li, DME, arenes, and their hybrid systems. We consider the ability of single Li and double Li to bind to solvent components, arenes, or DME. The results indicate that Li is more likely to bind with arenes than with DME (*E*_b_ ∼ −0.3 eV) to form Li-arene (*E*_b_ **∼** −1 eV) or Li_2_-arene (*E*_b_ **∼** −1.9 eV) systems. Mulliken charge analysis (Figure S12) indicated significant charge transfer from Li to arene to form the [Li^+^-(arene)^−^] complex, leading to much larger binding energies. Li^+^ can then be easily solvated by DME molecules with higher binding energies (Figure S13). The theoretical simulations are consistent with the observations in Figure S1, which shows that Li metal can be dissolved in the Li+BP system in the absence of sulfur. On the other hand, the DME solvent molecule cannot effectively stabilize the Li atom since both are electron-donating (Figure S12). Therefore, Li metal cannot dissolve directly in the DME solvent in the absence of sulfur, but the dissolution may be greatly enhanced by arene molecules, as observed in the experiments. When the Li atom is subsequently charged to Li^+^, it can be stabilized by the abundant lone pair electrons of the DME molecule, resulting in a more negative *E*_b_ than that of the electron-accepting arenes (Figure S13).

We then consider the competition of the adsorption of soluble LiPSs (Li_2_S_8_, Li_2_S_6_, and Li_2_S_4_) on arenes or DME solvent molecules. The geometries and average adsorption energies (*E̅*_ads_) are shown in Figure 8, where two types of adsorption of LiPSs, horizontal (*h*) and perpendicular (*p*), are both calculated. The results show that the *E̅*_ads_ of LiPSs on arenes are almost half (∼ −0.35 eV) of the *E̅*_ads_ of LiPSs on DME (∼ −0.65 eV). This may indicate that LiPSs can be better solvated by DME molecules than by the arenes in the solution. Accordingly, the reaction free energies (Δ*G*) of the Li-arene-DME systems reacting with S_8_ are calculated via eqs 11 and 12. All of the reactions are highly exothermic (<−4 eV) and readily take place in the solution. Based on these results, we propose that arenes, with their greater affinity for the Li atom, play a critical role in the process of Li metal dissolution and do not interfere much with the subsequent disproportionation mechanism during the formation of soluble LiPSs in LSBs.

11 $$2\mathrm{Li}\left(\mathrm{BP}\right)\left(\mathrm{DME}\right)+S_{8}\to {\mathrm{Li}}_{2}S_{8}{\left(\mathrm{DME}\right)}_{2}+2\mathrm{BP},\Delta G=-4.67\;\mathrm{eV}$$

12 $$2\mathrm{Li}\left(\mathrm{Naph}\right)\left(\mathrm{DME}\right)+S_{8}\to {\mathrm{Li}}_{2}S_{8}{\left(\mathrm{DME}\right)}_{2}+2\mathrm{Naph},\Delta G=-4.50\;\mathrm{eV}$$

**Figure 8:**
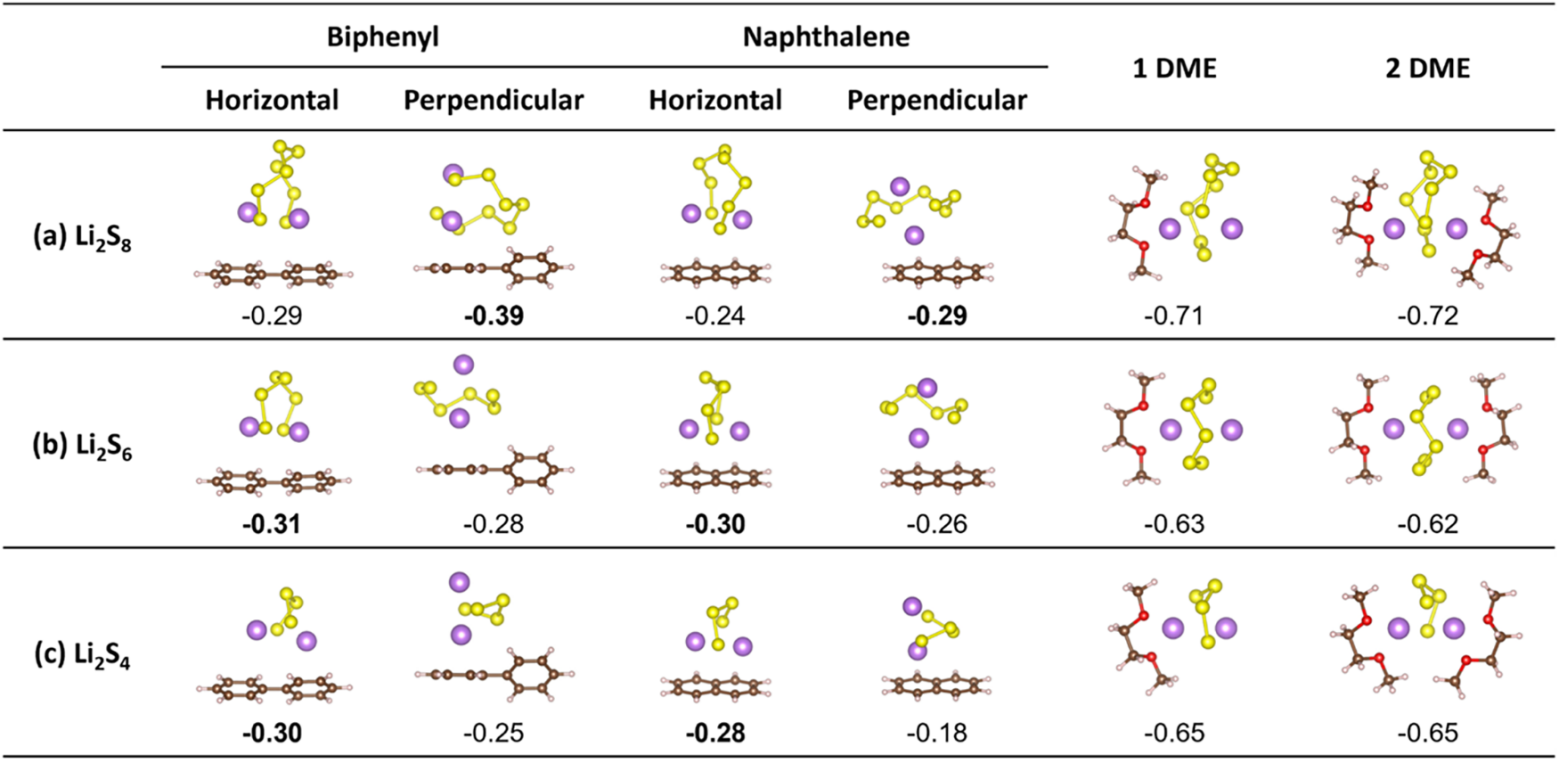
Geometries and average adsorption energies (*E̅*_ads_, in eV) of arene or DMEs on soluble (a) Li_2_S_8_, (b) Li_2_S_6_, and (c) Li_2_S_4_ molecules. The colors brown, white, yellow, purple, and red represent elements C, H, S, Li, and O, respectively.

## 4 Conclusions

In this study, the solubility, dissolution kinetics, and electrochemical performance of Li+BP+S and Li_2_S+S polysulfide systems are systematically evaluated to address key challenges for LSBs. The results demonstrate that the Li+BP+S system outperforms the Li_2_S+S system, exhibiting higher solubility (up to 12 M active sulfur), faster dissolution rates, and superior electrochemical stability. These improvements stem from the role of BP in facilitating polysulfide formation, accelerating sulfur ring breakdown, and leading to more efficient catholyte processing.

Electrochemical evaluations confirmed that the Li+BP+S catholyte enables increased sulfur utilization and long-term cycling stability. At an E/S ratio of 31.25 μL mg_S_^–1^, the system retains 927.8 mAh g^–1^ after 500 cycles, with a minimal capacity fade (0.03% per cycle). Even under lean electrolyte conditions (E/S = 7.8 μL mg_S_^–1^), it maintains over 100% capacity after 200 cycles, demonstrating excellent electrolyte utilization and reversibility. Additionally, it delivers a superior rate performance, recovering the initial capacity after cycling at high current densities.

Comprehensive material characterization (UV–Vis, ^7^Li NMR, Raman, and XRD) revealed that BP effectively inhibits LiPS clustering, enhances Li^2+^ transport, and promotes uniform sulfur distribution on the CNT electrode, contributing to stable catholyte–electrode interfaces. Postcycling analyses confirmed reduced sulfur agglomeration and improved deposition uniformity. Theoretical modeling further indicated that BP stabilizes lithium dissolution without disrupting polysulfide conversion mechanisms, reinforcing its critical role in catholyte design.

In summary, the Li+BP+S system presents a scalable and high-performance catholyte formulation that enhances solubility, processability, and electrochemical performance, making it a promising candidate for next-generation high-energy-density LSBs. Future work will focus on optimizing the catholyte composition to accelerate redox kinetics and minimize parasitic reactions, as well as developing engineered cathode architectures to maximize sulfur utilization and power density under lean electrolyte conditions. In parallel, addressing integration challenges such as the safe handling of metallic lithium and large-scale processing of reactive components will be essential for transitioning this technology toward commercial LSB systems.

## Supplementary Material



## Notes

Computational details, polysulfide synthesis and solubility data, electrochemical performance of modified and aged catholytes, XPS spectra and depth profiling, DFT-optimized structures, and a benchmarking table of reported polysulfide catholytes (PDF)
