# Supplementary material for: Transformation of Polysulfide Catholyte Chemistry through Lithium-Arene Complexes for Superior Solubility and Cyclability in Li–S Batteries
Source: JACS Au. 2025 Jul 9;5(8):3866–78. doi: 10.1021/jacsau.5c00537 (PMC12381734; doi:10.1021/jacsau.5c00537)
Supplement: Supplementary file 1 [file au5c00537_si_001.pdf]

Supporting Information

Transformation of Polysulfide Catholyte Chemistry through Lithium-Arene Complexes for Superior Solubility and Cyclability in Li-S Batteries

Ngoc Long Le[1], *Sih-Ling Hsu*[2], *Thi Hang Vu*[1], *Chi-You Liu*[2,3], *Quang Huy Dinh*[1], *Avi Arya*[1], *Elise Yu-Tzu Li**[2] *and Yu-Sheng Su**[1,4]

[1]International College of Semiconductor Technology, National Yang Ming Chiao Tung University, 1001 Daxue Road, Hsinchu 300093, Taiwan

[2]Department of Chemistry, National Taiwan Normal University, No. 88, Section 4, Tingzhou Road, Taipei 11677, Taiwan

[3]Department of Chemistry, Soochow University, No. 70, Linhsi Road, Taipei 11102, Taiwan

[4]Industry Academia Innovation School, National Yang Ming Chiao Tung University, 1001 Daxue Road, Hsinchu 300093, Taiwan

Computational Details.

The dispersion-corrected hybrid functionals wB97XD and the basis set 6-311+G (d, p) are selected to calculate the LiPS, arene, Li-arene, and the explicitly solvated systems using the G16 software.[R1] The implicit solvation model, integral equation formalism polarizable continuum model (IEFPCM), is included and the parameter of tetrahydrofuran (ε = 7.43) is chosen to mimic the experimental DOL:DME solution environment (ε ~ 7.2). The spin polarization of the unpaired electrons is considered by the fragmentation settings. The convergence criteria for the self-consistent field iteration steps are set to 10^{-8} eV (scf = tight). The binding energies ($\boldsymbol{E_b}$, in eV) and the average adsorption energies ($\boldsymbol{\bar{E}_{ads}}$, in eV) are calculated using the following equations, respectively:

$$E_b = E_{tot} - E_{components} \qquad \textbf{(Equation S1)}$$

$$\bar{E}_{ads} = \frac{E_{tot} - E_{adsorbates}}{n} \qquad \textbf{(Equation S2)}$$

where the E_{tot}, $E_{components}$, and $E_{adsorbate(s)}$ represent the energies of the total systems, the reactant components, and the adsorbates (Li, Li^+, LiPSs, DME, arenes), respectively. The n represents the number of the adsorbates. The free energies are calculated at a temperature of 298K.

Reference

[R1] M. J. Frisch, G. W. Trucks, H. B. Schlegel, G. E. Scuseria, M. A. Robb,J. R. Cheeseman, G. Scalmani, V. Barone, G. A. Petersson,H. Nakatsuji, X. Li, M. Caricato, A. V. Marenich, J. Bloino,B. G. Janesko, R. Gomperts, B. Mennucci, H. P. Hratchian,J. V. Ortiz, A. F. Izmaylov, J. L. Sonnenberg, D. Williams-Young,F. Ding, F. Lipparini, F. Egidi, J. Goings, B. Peng, A. Petrone,T. Henderson, D. Ranasinghe, et al., Gaussian 16, Revision A.01, **2016**.

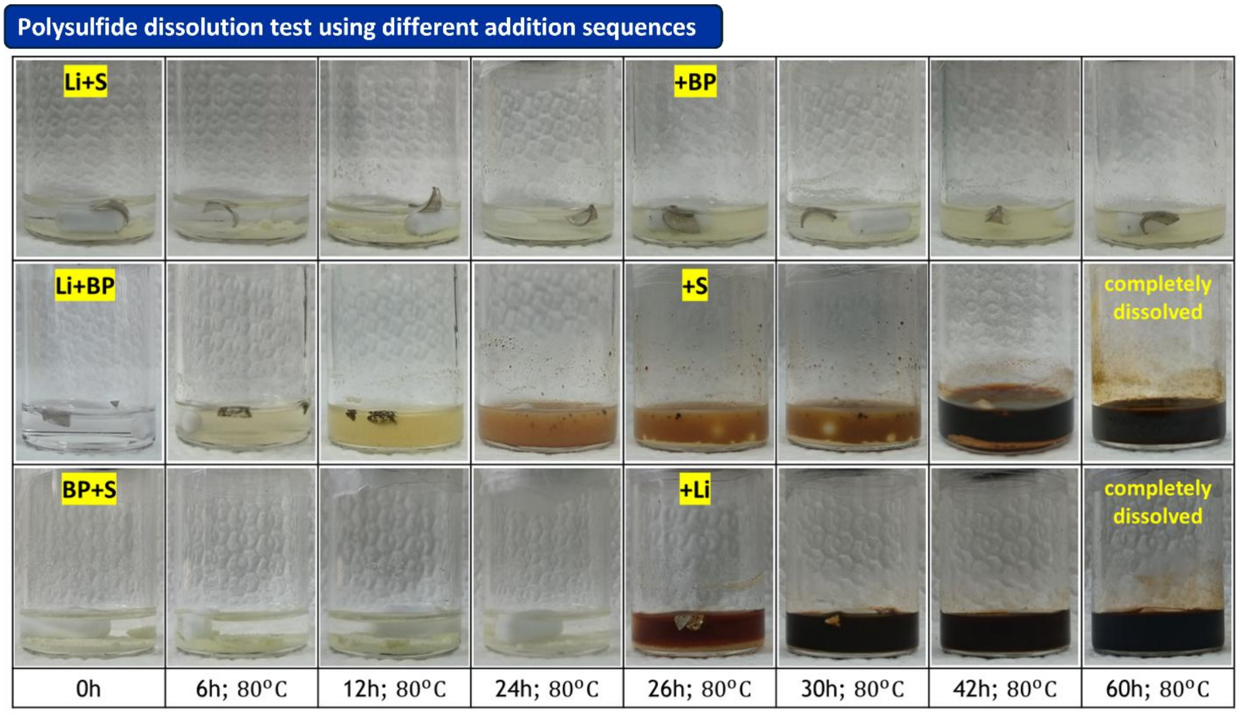

Figure S1. Comparison of synthetic procedures for polysulfide solutions with different addition sequences of chemicals in the 1.85 M electrolyte.

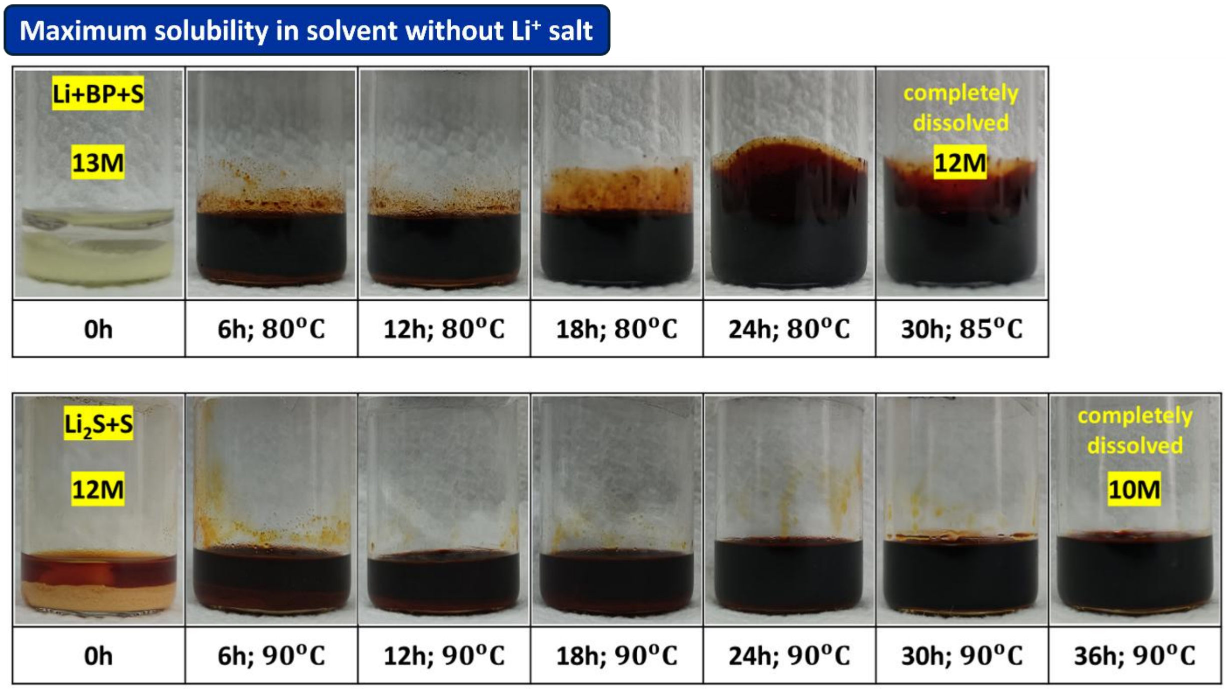

Figure S2. Maximum solubility of polysulfide solutions in DOL/DME solvent without lithium salt.

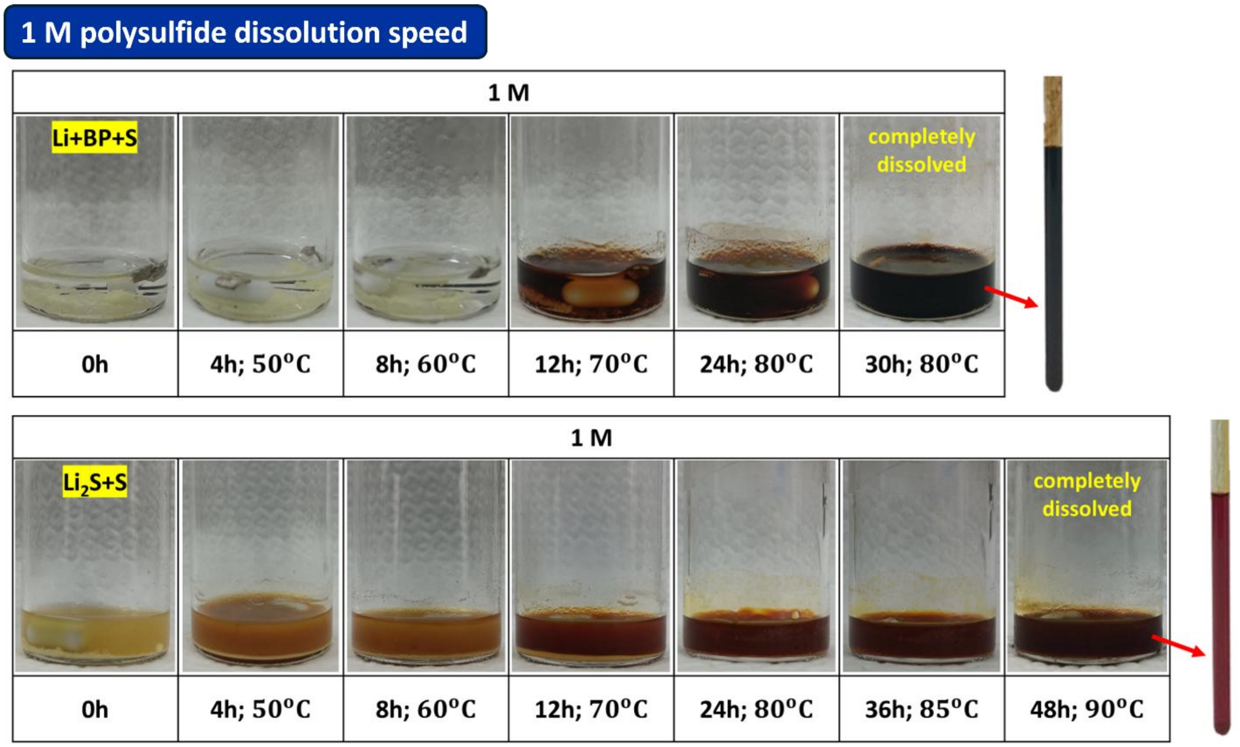

Figure S3. Dissolution behavior of polysulfide solutions containing 1 M active sulfur in the 1.85 M electrolyte.

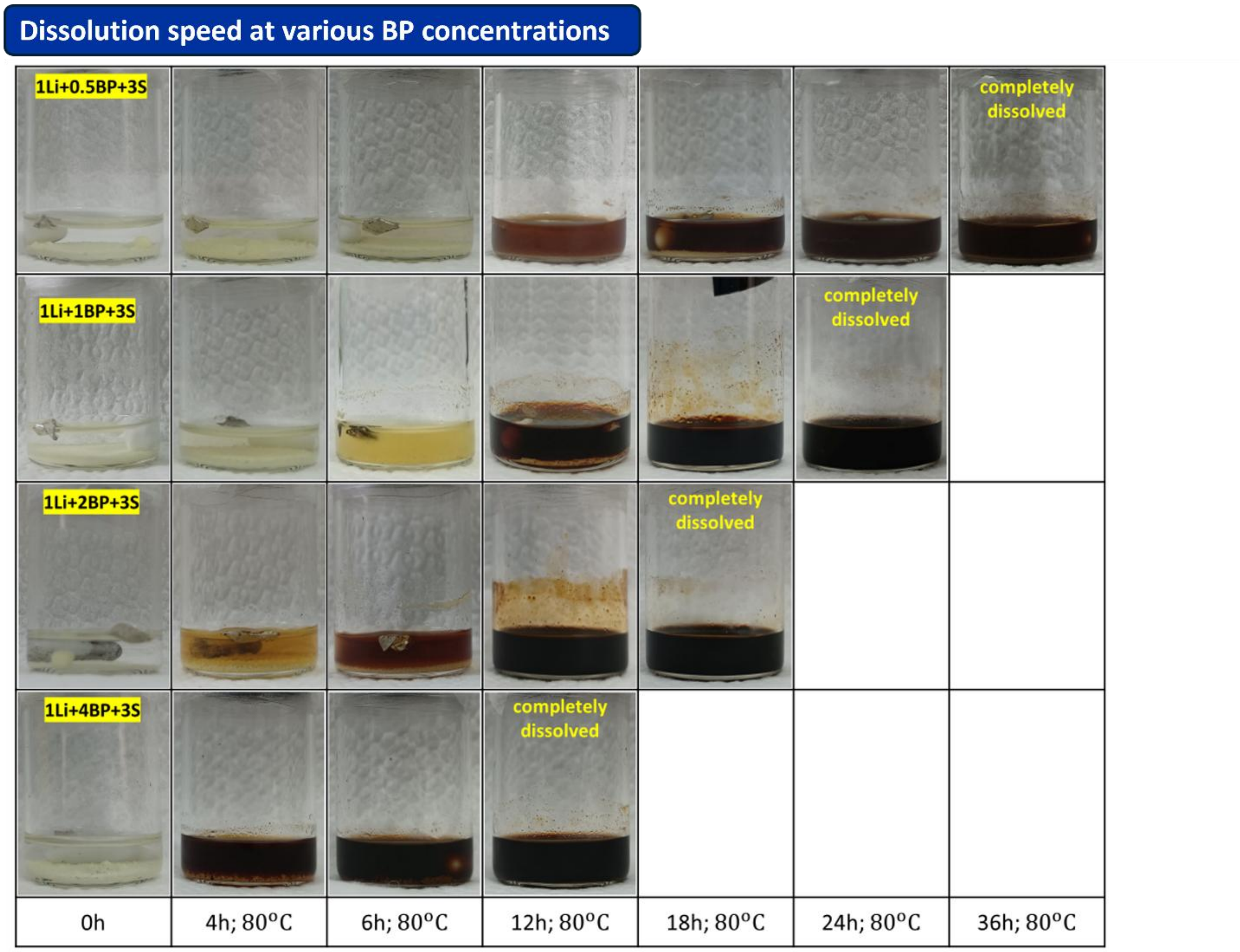

Figure S4. Dissolution behavior of polysulfide solutions containing 1 M active sulfur in the 1.85 M electrolyte with varying BP concentrations.

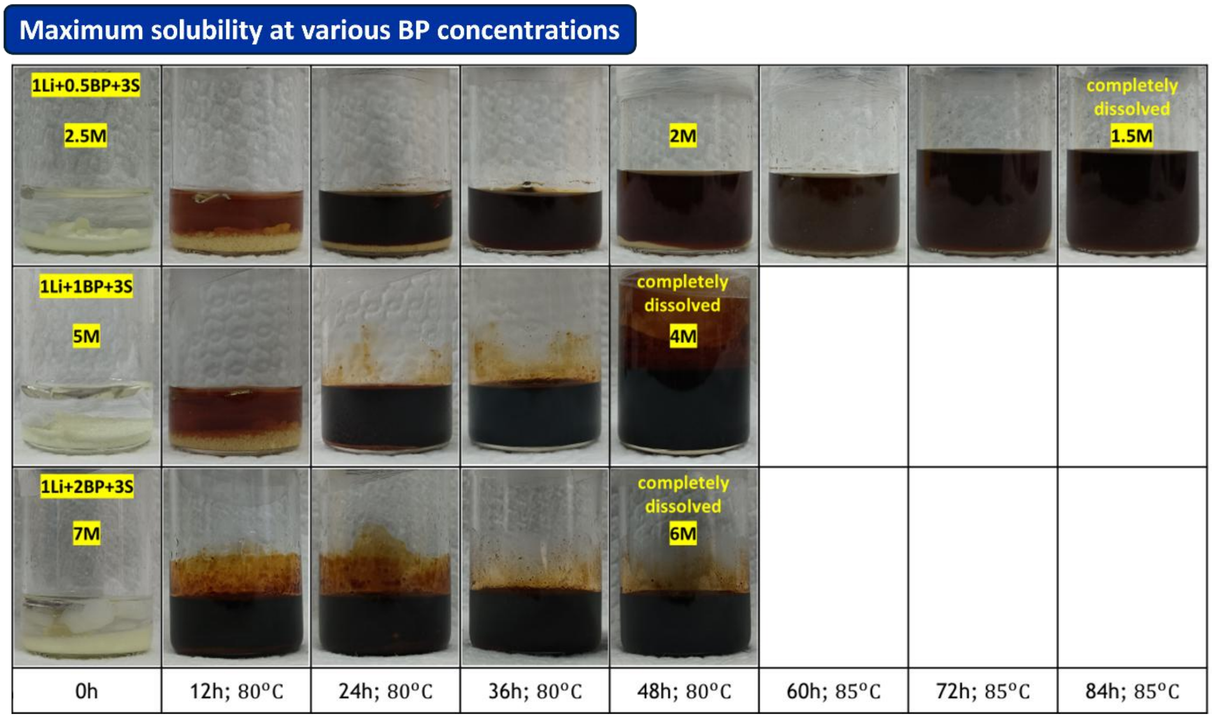

Figure S5. Maximum solubility of polysulfide solutions in the 1.85 M electrolyte with varying BP concentrations.

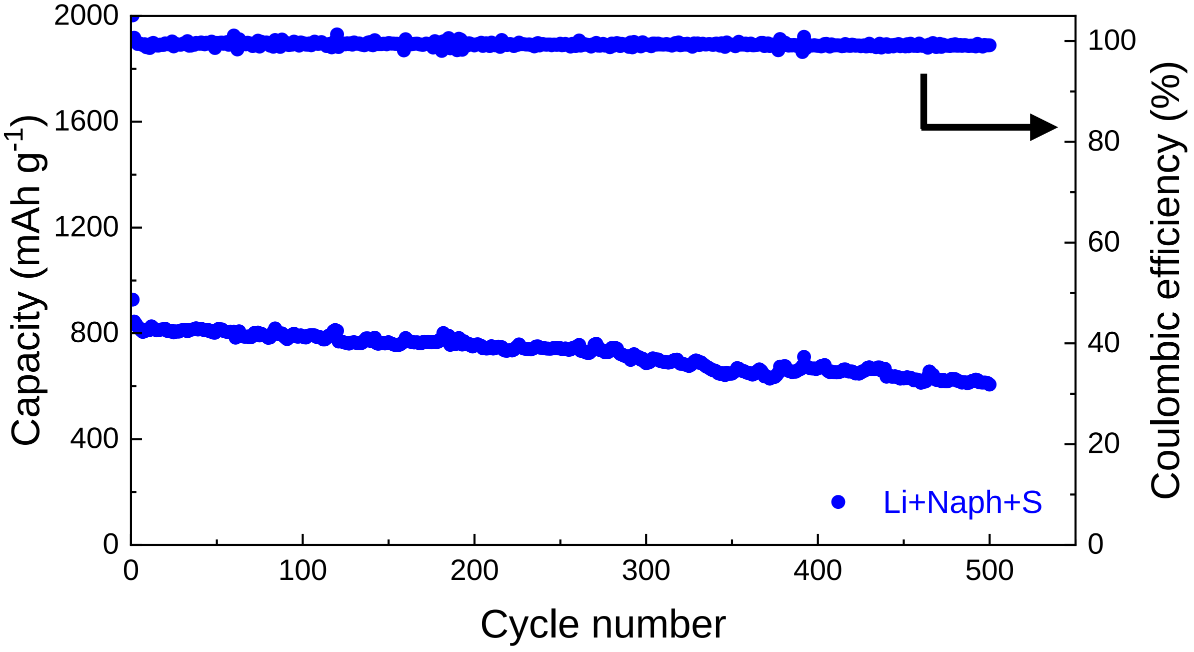

Figure S6. Cycling performance of the Li+Naph+S polysulfide catholyte at 0.5C.

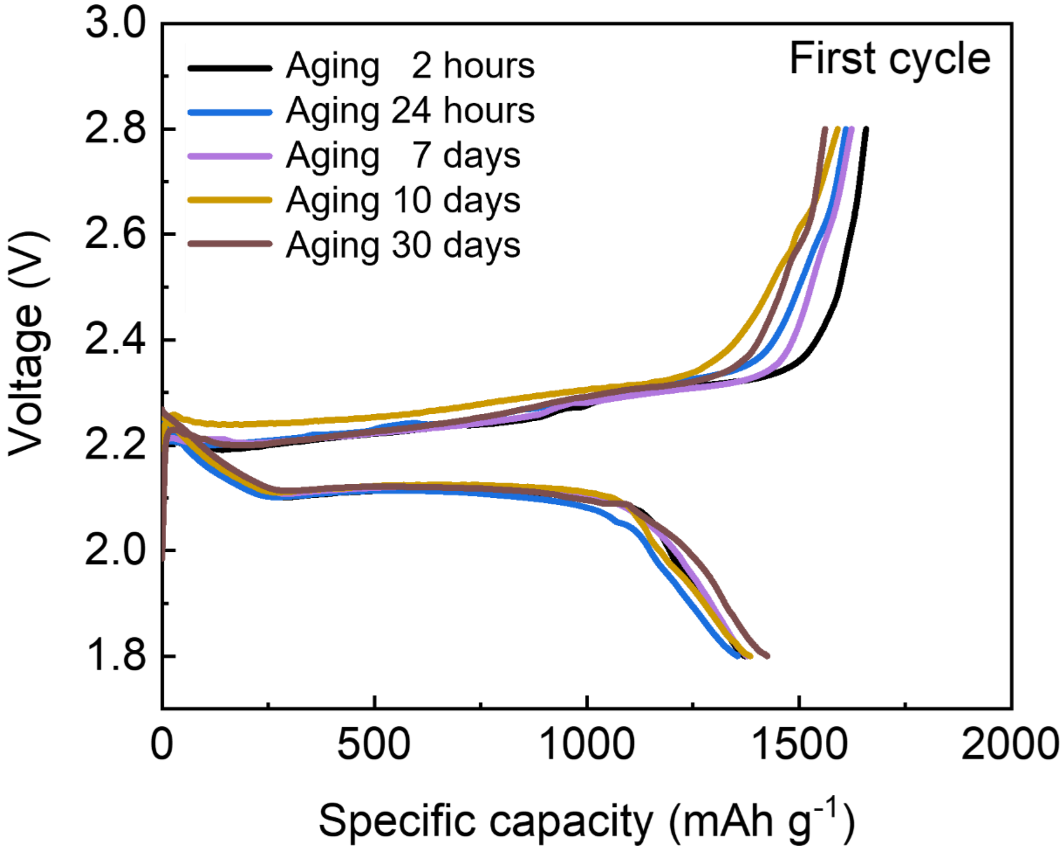

Figure S7. Charge/discharge profiles of the Li+BP+S polysulfide catholyte at 0.1 C under different aging conditions, demonstrating its stability and capacity retention over time.

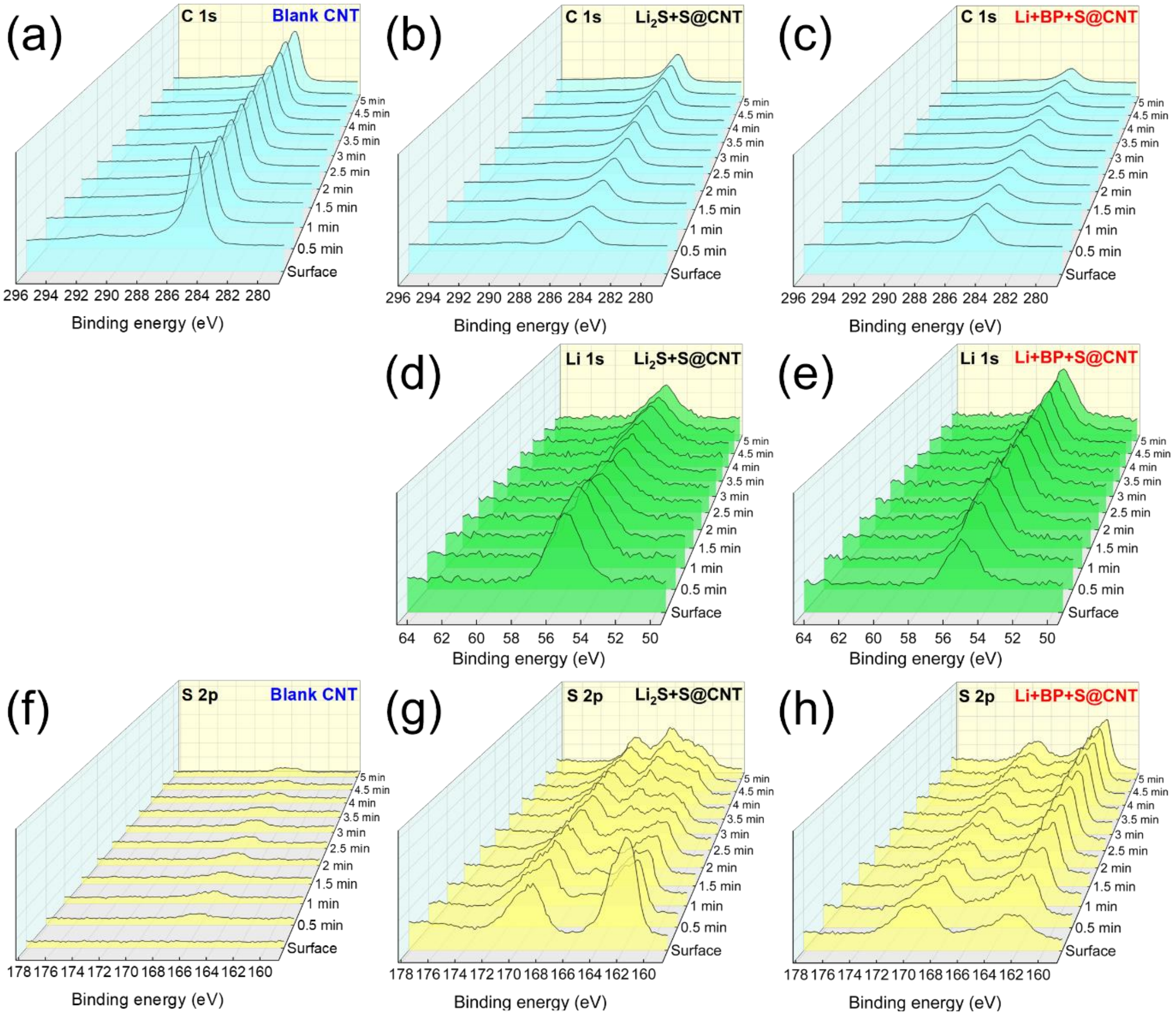

Figure S8. XPS depth profiles of blank CNT, Li_2S+S@CNT, and Li+BP+S@CNT electrodes after 100 cycles with varying etching times. XPS spectra of (a–c) C 1s, (d, e) Li 1s, and (f–h) S 2p signals at different etching depths.

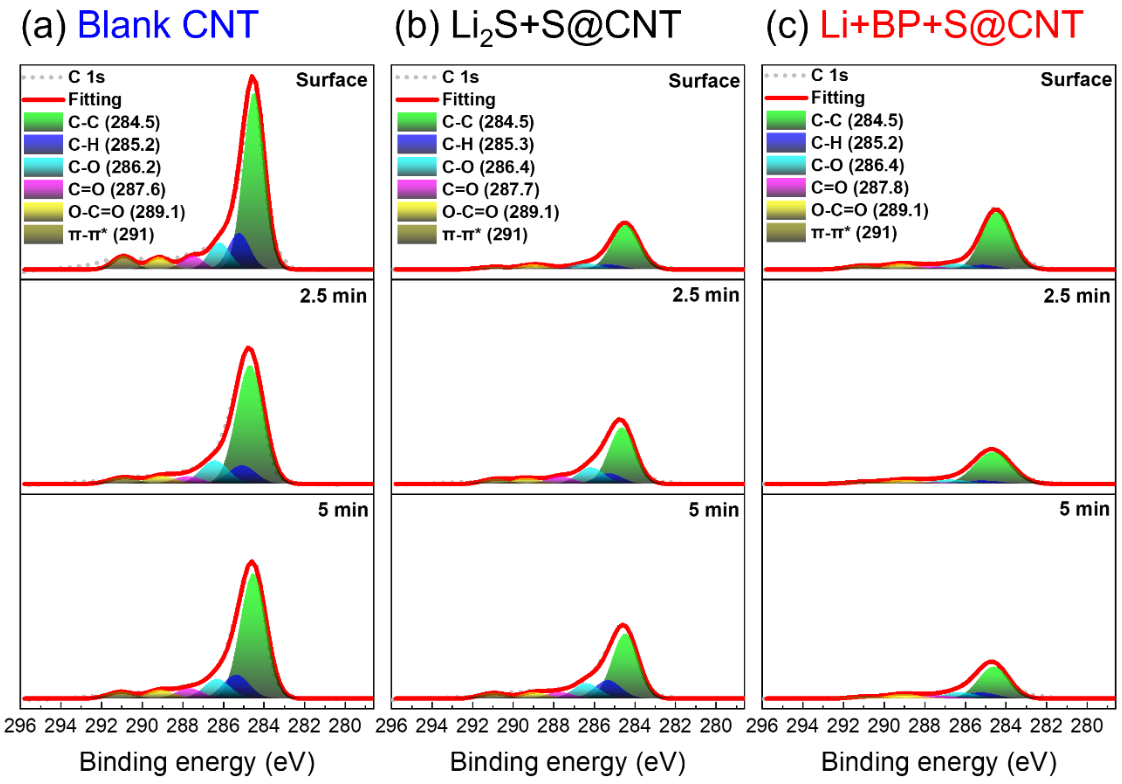

Figure S9. XPS spectra of the C 1s peak for (a) blank CNT, (b) Li_2S+S@CNT, and (c) Li+BP+S@CNT electrodes after 100 cycles, with corresponding deconvoluted fitted peaks.

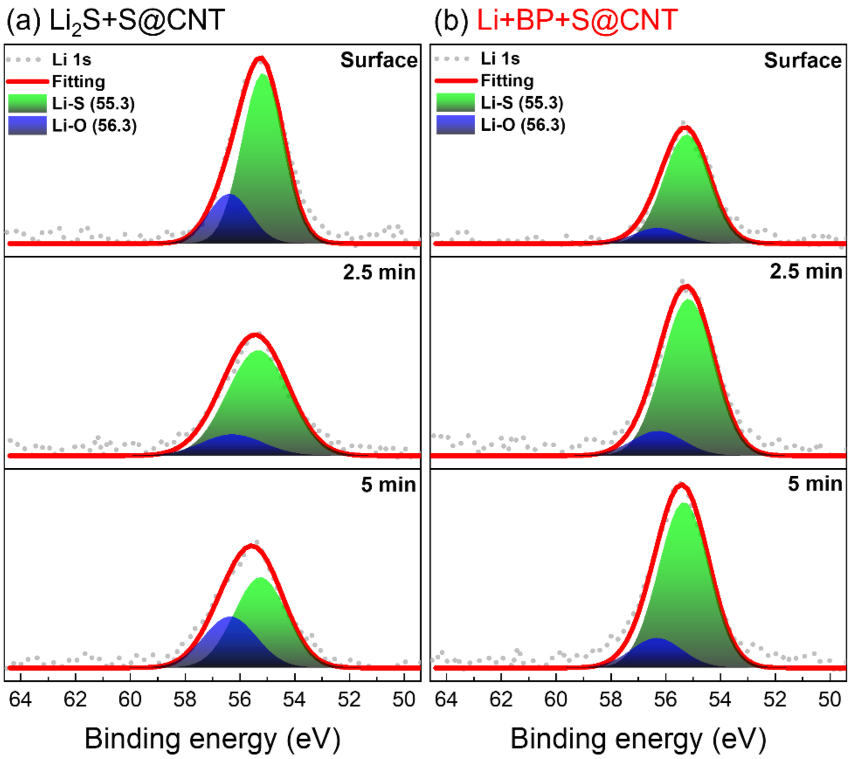

Figure S10. XPS spectra of the Li 1s peak for (a) Li_2S+S@CNT and (b) Li+BP+S@CNT electrodes after 100 cycles, along with their deconvoluted fitted peaks at different etching depths.

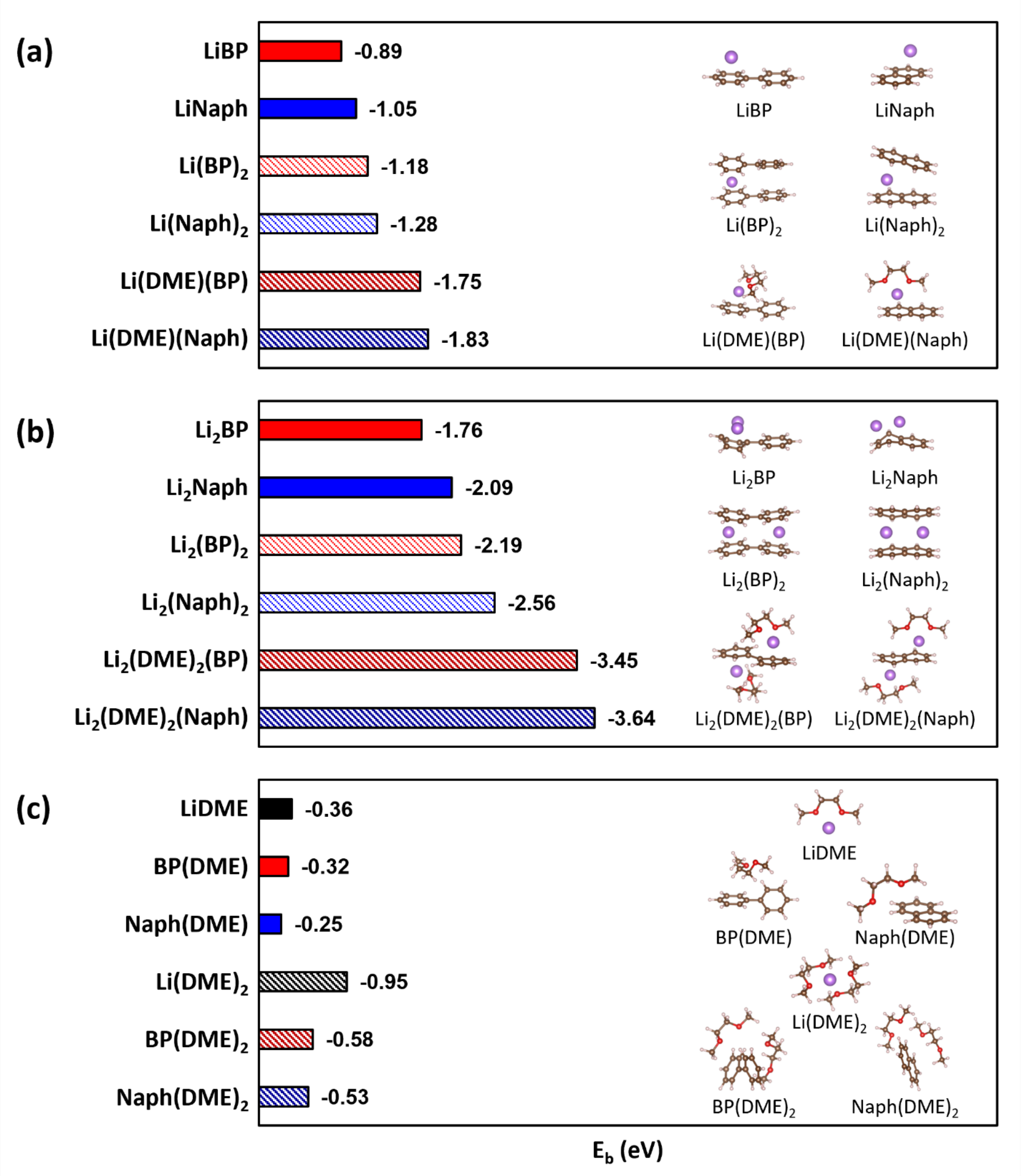

Figure S11. The optimized geometries, the binding energies ($\boldsymbol{E_b}$, in eV) of Li, arene, and DME as reactants in (a) single Li-, (b) double Li-, and (c) solvent-containing systems. The colors brown, white, purple, and red represent the elements C, H, Li, and O, respectively.

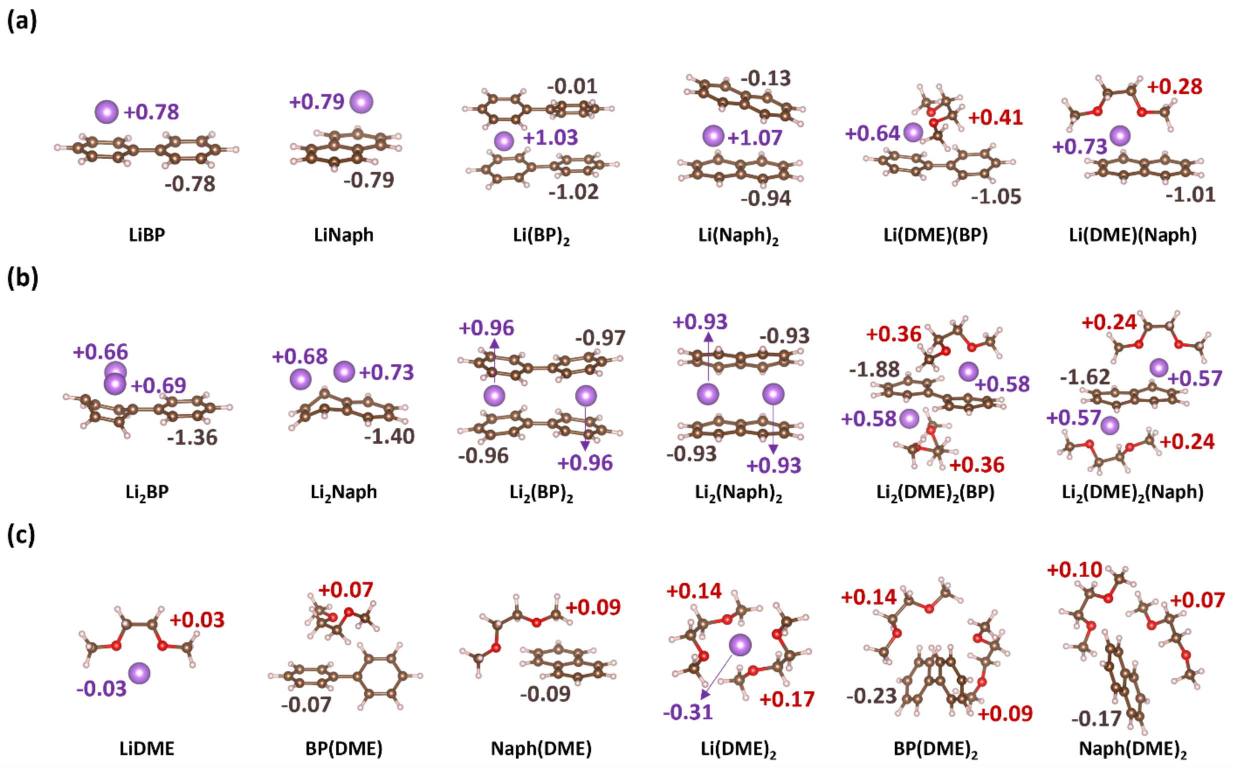

Figure S12. Optimized structures and the Mulliken charge results (*q*, in |e|) of (a) single Li and (b) double Li containing systems. Other related systems are shown in (c). The colored atoms brown, white, purple, and red represent the elements C, H, Li, and O, respectively. The colored value purple, black, and red represent the Mulliken charge of Li, arene, and DME, respectively.

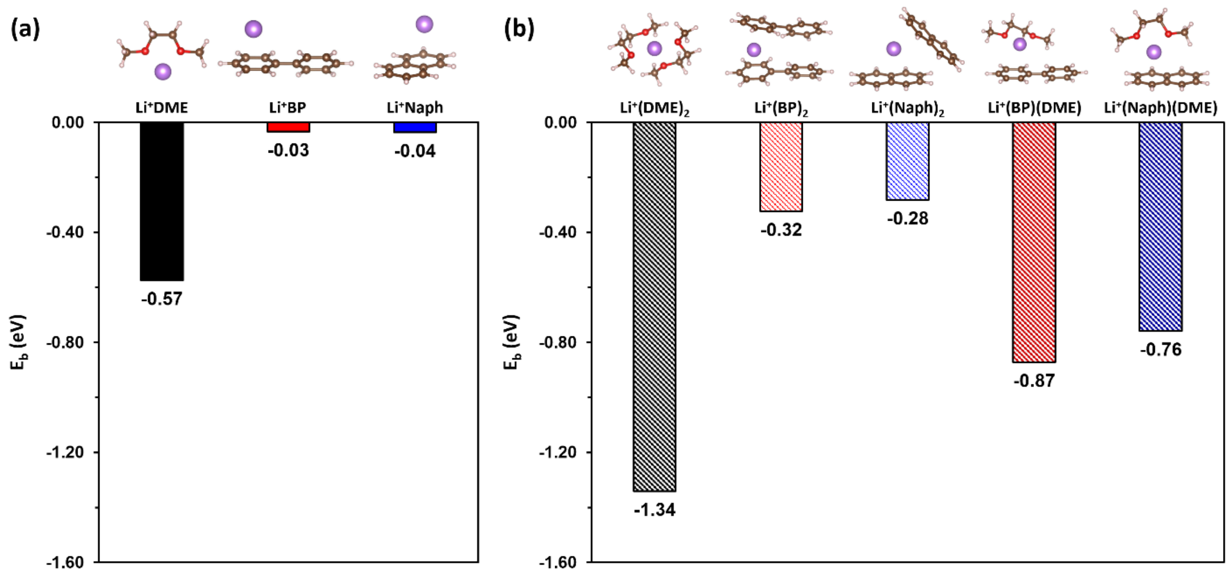

Figure S13. The geometries and the binding energies ($\boldsymbol{E_b}$, in eV) of (a) two- and (b) three-component combinations, constructed from arenes, DME, and Li^+. The colors brown, white, purple, and red represent the elements C, H, Li, and O, respectively.

Table S1. Comparison of various polysulfide catholytes in Li-S batteries.

**Catholyte Design**	**Conc.**	**Temp. (ºC)**	**Duration**	**Cathode Design**	**S loading (mg cm^{-2})**	**E/S ratio (µL mg^{-1})**	**Capacity (mAh g^{-1})**	**Capacity Fade per Cycle (%)**	**Cycle Rate**	**Ref.**
**Li_2S+S (Li_2S_8)**	0.013 M	80	overnight	CC/1T-MoS_2	2.1<br>4.4	7.6<br>3.7	1238<br>1176	0.05<br>0.08	0.5C	1
**Li_2S+S (Li_2S_6)**	1 M	60	48h	$MoTe_2$@Graphene/ CC	1<br>6	15<br>5	1263<br>787	0.013<br>0.089	0.2C<br>0.1C	2
**Li_2S+S (Li_2S_6)**	0.52 M	70	48h	rGO sponge	2.2<br>6.6	N/A	1607<br>1330	0.032<br>0.064	1C	3
**Li_2S+S (Li_2S_6)**	0.5 M	50	N/A	Cu_2ZnSnS_4/CC	1.0–2.0	N/A	1200	0.058	0.5 mA cm^{-2}	4
**Li_2S+S (Li_2S_8)**	0.5 M	RT	24	rGO aerogel	3.2	N/A	~1063	0.04	0.1C	5
**Li_2S+S (Li_2S_4)**	N/A	45	N/A	CNT foam	N/A	4.4	536	0.13	0.4 mA cm^{-2}	6
**Li_2S–P_2S_5**	N/A	50	2h	Comercial CC	3.75	10	730	0.51	0.1C	7
**Li+BP+S (Li_2S_6)**	1 M	80	30h	CNT paper	0.64<br>1.45<br>2.17	31.25<br>10.42<br>7.8	1116<br>633<br>578	0.03<br>0.07<br>0	0.5C	This work

References

(1) Wang, M.; Yang, H.; Shen, K.; Xu, H.; Wang, W.; Yang, Z.; Zhang, L.; Chen, J.; Huang, Y.; Chen, M.; Mitlin, D.; Li, X. Stable Lithium Sulfur Battery Based on In Situ Electrocatalytically Formed Li_2S on Metallic MoS_2–Carbon Cloth Support. *Small Methods* **2020**, *4* (9), 2000353. https://doi.org/10.1002/smtd.202000353.

(2) Wei, Z.; Sarwar, S.; Azam, S.; Ahasan, M. R.; Voyda, M.; Zhang, X.; Wang, R. Ultrafast Microwave Synthesis of $MoTe_2$@graphene Composites Accelerating Polysulfide Conversion and Promoting Li_2S Nucleation for High-Performance Li-S Batteries. *Journal of Colloid and Interface Science* **2023**, *635*, 391–405. https://doi.org/10.1016/j.jcis.2022.12.111.

(3) Chiochan, P.; Kosasang, S.; Ma, N.; Duangdangchote, S.; Suktha, P.; Sawangphruk, M. Confining Li_2S_6 Catholyte in 3D Graphene Sponge with Ultrahigh Total Pore Volume and Oxygen-Containing Groups for Lithium-Sulfur Batteries. *Carbon* **2020**, *158*, 244–255. https://doi.org/10.1016/j.carbon.2019.12.015.

(4) Zha, C.; Wu, D.; Zhao, Y.; Deng, J.; Wu, J.; Wu, R.; Yang, M.; Wang, L.; Chen, H. Two-Dimensional Multimetallic Sulfide Nanosheets with Multi-Active Sites to Enhance Polysulfide Redox Reactions in Liquid Li_2S_6-Based Lithium-Polysulfide Batteries. *Journal of Energy Chemistry* **2021**, *52*, 163–169. https://doi.org/10.1016/j.jechem.2020.04.059.

(5) Cavallo, C.; Agostini, M.; Genders, J. P.; Abdelhamid, M. E.; Matic, A. A Free-Standing Reduced Graphene Oxide Aerogel as Supporting Electrode in a Fluorine-Free Li2S8 Catholyte Li-S Battery. *Journal of Power Sources* **2019**, *416*, 111–117. https://doi.org/10.1016/j.jpowsour.2019.01.081.

(6) Shen, C.; Xie, J.; Zhang, M.; Andrei, P.; Zheng, J. P.; Hendrickson, M.; Plichta, E. J. A Li-$Li2S_4$ Battery with Improved Discharge Capacity and Cycle Life at Low Electrolyte/Sulfur Ratios. *Journal of Power Sources* **2019**, *414*, 412–419. https://doi.org/10.1016/j.jpowsour.2019.01.029.

(7) Yen, Y.-J.; Chung, S.-H. A Li_2S-Based Catholyte/Solid-State-Electrolyte Composite for Electrochemically Stable Lithium–Sulfur Batteries. *ACS Appl. Mater. Interfaces* **2021**, *13* (49), 58712–58722. https://doi.org/10.1021/acsami.1c18871.
